# Sex Differences in Genetic Architecture of Complex Phenotypes?

**DOI:** 10.1371/journal.pone.0047371

**Published:** 2012-12-18

**Authors:** Jacqueline M. Vink, Meike Bartels, Toos C. E. M. van Beijsterveldt, Jenny van Dongen, Jenny H. D. A. van Beek, Marijn A. Distel, Marleen H. M. de Moor, Dirk J. A. Smit, Camelia C. Minica, Lannie Ligthart, Lot M. Geels, Abdel Abdellaoui, Christel M. Middeldorp, Jouke Jan Hottenga, Gonneke Willemsen, Eco J. C. de Geus, Dorret I. Boomsma

**Affiliations:** Netherlands Twin Register, Department of Biological Psychology, VU University Amsterdam, Amsterdam, The Netherlands; The George Washington University, United States of America

## Abstract

We examined sex differences in familial resemblance for a broad range of behavioral, psychiatric and health related phenotypes (122 complex traits) in children and adults. There is a renewed interest in the importance of genotype by sex interaction in, for example, genome-wide association (GWA) studies of complex phenotypes. If different genes play a role across sex, GWA studies should consider the effect of genetic variants separately in men and women, which affects statistical power. Twin and family studies offer an opportunity to compare resemblance between opposite-sex family members to the resemblance between same-sex relatives, thereby presenting a test of quantitative and qualitative sex differences in the genetic architecture of complex traits. We analyzed data on lifestyle, personality, psychiatric disorder, health, growth, development and metabolic traits in dizygotic (DZ) same-sex and opposite-sex twins, as these siblings are perfectly matched for age and prenatal exposures. Sample size varied from slightly over 300 subjects for measures of brain function such as EEG power to over 30,000 subjects for childhood psychopathology and birth weight. For most phenotypes, sample sizes were large, with an average sample size of 9027 individuals. By testing whether the resemblance in DZ opposite-sex pairs is the same as in DZ same-sex pairs, we obtain evidence for genetic qualitative sex-differences in the genetic architecture of complex traits for 4% of phenotypes. We conclude that for most traits that were examined, the current evidence is that same the genes are operating in men and women.

## Introduction

Heritability is defined as the ratio of the genetic variance over the total variance of a trait [1], and can differ between the sexes for multiple reasons. Different genes can be expressed in men and women, but even when the same genes are expressed in both sexes their relative importance can differ, and the environmental variance can vary, thereby also changing the ratio of genetic over total variance. In a classical paper from 1978, Eaves *et al* suggested that the key to detection of sex by genotype interactions lies with **opposite-sex twin pairs** who should be comparable in their similarity with **dizygotic same-sex** (DZss) twin pairs if a similar mechanism is accounting for the variation in the trait in males and females [2]. To cite Eaves and colleagues: “*Many twin studies in the past have deliberately excluded unlike-sex twins, presumably out of a mistaken belief that concentrating on like-sex pairs ‘controls’ for the effect of sex. In reality, exactly the reverse is true. Omission of unlike-sex pairs removes the most important tool for the early identification of sex-dependent mechanisms of determination.*”

Resemblances among first degree relatives such as dizygotic twins or sibling pairs can be summarized by correlations (r). For phenotypes assessed on a continuous scale r can be a product-moment or intra-class correlation. For ordinal and dichotomous traits, r can be a polychoric or tetrachoric correlation, which summarizes the familial resemblance on the liability scale [3]. The expectations for sibling or DZ twins correlations assuming an autosomal inheritance pattern can be expressed as:
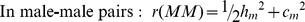


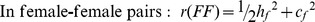


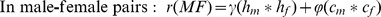
where h^2^ represents the narrow-sense heritability i.e. the additive genetic variance divided by the total phenotypic variance of the phenotype, and c^2^ gives the standardized common environmental variance shared by family members. In same-sex sibling pairs, under the assumption of random mating, h^2^ is weighted by 0.5 (e.g. Jacquard, 1974). In opposite-sex pairs, the genetic correlation between relatives is symbolized by γ. Common environmental factors, defined as all environmental factors that increase resemblance of relatives for non-genetic reasons, are correlated unity in same-sex relatives and φ is the correlation among environmental factors in male-female pairs. When γ<0.5 there is evidence for qualitative sex differences, i.e. for the hypothesis that different genes are expressed in men and women. When γ<0.5, the observed correlation in opposite-sex relatives will be lower than predicted from the resemblances in same-sex relatives. However, an alternative explanation for this observation might be that environmental sources of covariance between relatives differ in men and women. When φ<1, male-female pairs share fewer environmental factors than same-sex pairs.

In this contribution we consider the presence of sex differences in the genetic architecture of complex human traits by examining the resemblance for dizygotic same-sex (DZss) and opposite-sex (DZos) twin pairs for a large number of phenotypes that are currently studied in GWA consortia. The data come from the large, population-based Netherlands Twin Register that collects longitudinal data on lifestyle such as alcohol and nicotine use, personality, psychiatric disorder in children and in adults, health, development, cardiovascular risk factors and metabolic traits [4]–[7]. A total of 122 variables is included in the study, encompassing growth during childhood, anthropometric measures, brain function, IQ, personality, psychiatric disorders, migraine, cardiovascular and metabolic traits across a range of ages. Large sample sizes (exceeding 30.000 participants) are available for some traits.

To test for qualitative sex differences, we focus on dizygotic twin pairs since these siblings are perfectly matched for age, upbringing, and prenatal exposures. For all traits, the resemblance in monozygotic (MZ) twin pairs is also given to establish that familial resemblance is due to genetic factors rather than (or in addition to) shared environment. A first series of analyses is carried out to establish whether DZss and DZos twin pair correlations are the same. If the resemblance in DZos pairs is lower than in DZss pairs, we address the question whether the lower resemblance is due to different genes expressed in men and women or due to the fact that men and women share fewer environmental factors.

## Results

A detailed overview of the data including sample sizes and age at data collection is presented in Tables S1, S2, S3, S4, S5. Total sample sizes (including MZ and DZ twins) varied from slightly over 300 subjects for measures of brain function such EEG power, between 4000 and 7000 for personality, around 10,000 for smoking and drinking behavior in adults, between 10,000 and 20,000 for anthropometric traits to nearly 35,000 subjects for birth weight and over 30.000 individuals for indices of childhood psychopathology. For most variables, sample sizes were large, with an average sample size of 9027 and a median of 7223 individuals.

The Tables S1, S2, S3, S4, S5 also summarize the means and variances for continuous traits and the prevalences for categorical variables. Sex differences in means and prevalances were in line with previous reports, e.g. women score higher on depression/anxiety and more often have migraine. Men are taller, score higher on sensation seeking scales, more often use cannabis (adults), tend to smoke more often, drink more coffee and more often report alcohol problems. In children, aggressive and attention problems occur more often in boys, while somatic complaints are more often seen in girls.

**Figure 1:**
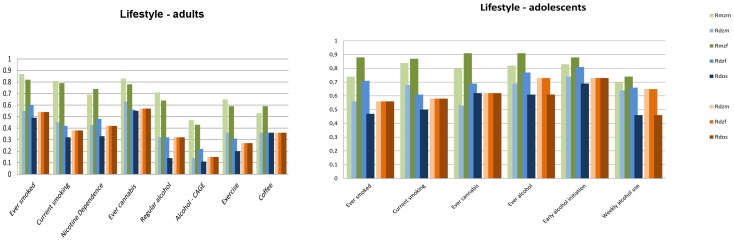
Twin correlations for Lifestyle variables. 1A: adult twins and 1B: adolescent twins. The green bars represent correlations in Monozygotic Male (MZM) twin pairs (light green) and Monozygotic Female (MZF) twin pairs. The blue bars represent correlations in Dizygotic Male (DZM) twin pairs, Dizygotic Female (DZF) twin pairs and Dizygotic Opposite Sex (DOS) twin pairs. The green and blue bars reflect the correlations in a full model, while the orange bars reflect twin correlations for Dizygotic (DZ) twin pairs in the most parsimonious model, with: DZM (light orange), MZF (normal orange), DOS (dark orange).

**Figure 2:**
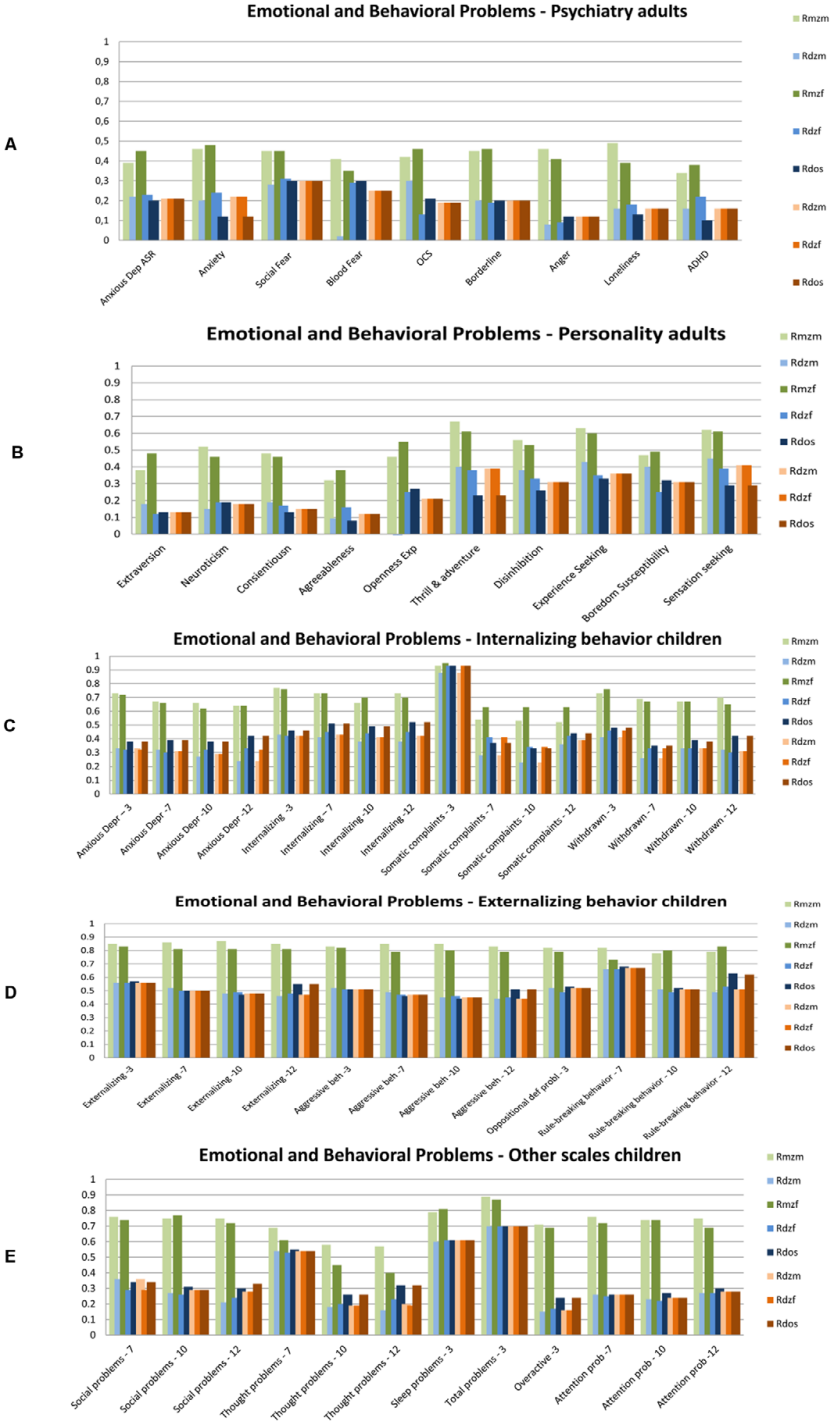
Twin correlations for Behavioral and Emotional Problems. 1A: adult psychiatry, 1B: adult psychology, 1C Internalizing behavior in children, 1D Externalizing behavior in children, 1E other scales in children. The green bars represent correlations in Monozygotic Male (MZM) twin pairs (light green) and Monozygotic Female (MZF) twin pairs. The blue bars represent correlations in Dizygotic Male (DZM) twin pairs, Dizygotic Female (DZF) twin pairs and Dizygotic Opposite Sex (DOS) twin pairs. The green and blue bars reflect the correlations in a full model, while the orange bars reflect twin correlations for Dizygotic (DZ) twin pairs in the most parsimonious model, with: DZM (light orange), MZF (normal orange), DOS (dark orange).

**Figure 3:**
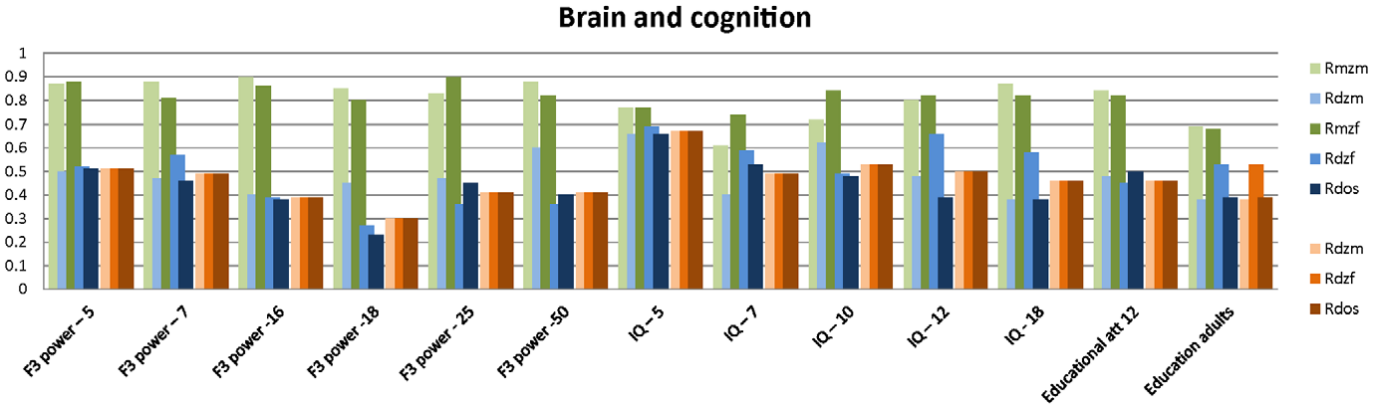
Twin correlations for Brain and Cognition variables. The green bars represent correlations in Monozygotic Male (MZM) twin pairs (light green) and Monozygotic Female (MZF) twin pairs. The blue bars represent correlations in Dizygotic Male (DZM) twin pairs, Dizygotic Female (DZF) twin pairs and Dizygotic Opposite Sex (DOS) twin pairs. The green and blue bars reflect the correlations in a full model, while the orange bars reflect twin correlations for Dizygotic (DZ) twin pairs in the most parsimonious model, with: DZM (light orange), MZF (normal orange), DOS (dark orange).

**Figure 4:**
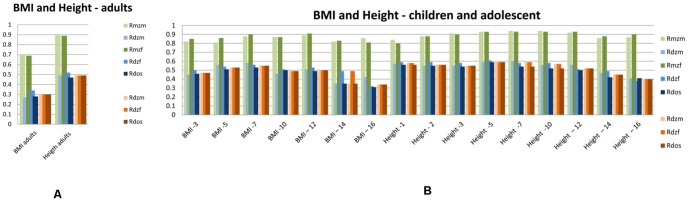
Twin correlations for BMI and Height. 1A: adult twins and 1B: adolescent twins. The green bars represent correlations in Monozygotic Male (MZM) twin pairs (light green) and Monozygotic Female (MZF) twin pairs. The blue bars represent correlations in Dizygotic Male (DZM) twin pairs, Dizygotic Female (DZF) twin pairs and Dizygotic Opposite Sex (DOS) twin pairs. The green and blue bars reflect the correlations in a full model, while the orange bars reflect twin correlations for Dizygotic (DZ) twin pairs in the most parsimonious model, with: DZM (light orange), MZF (normal orange), DOS (dark orange).

**Figure 5:**
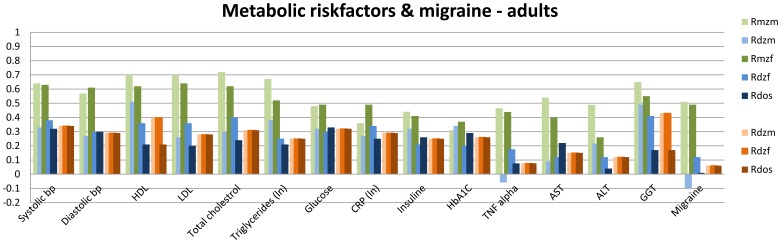
Twin correlations for Metabolic risk factors and Migraine. The green bars represent correlations in Monozygotic Male (MZM) twin pairs (light green) and Monozygotic Female (MZF) twin pairs. The blue bars represent correlations in Dizygotic Male (DZM) twin pairs, Dizygotic Female (DZF) twin pairs and Dizygotic Opposite Sex (DOS) twin pairs. The green and blue bars reflect the correlations in a full model, while the orange bars reflect twin correlations for Dizygotic (DZ) twin pairs in the most parsimonious model, with: DZM (light orange), MZF (normal orange), DOS (dark orange).

Figures 1, 2, 3, 4, 5 summarize the resemblances for MZ and DZ twin pairs across 5 major domains; *Lifestyle*, including smoking behavior, use of soft drugs, alcohol use and abuse, and exercise and sports behavior (Figure 1A en 1B); *Emotional and Behavioral Problems*, including psychiatric measures (e.g. depression, borderline, phobia, ADHD) in adults (Figure 2A), personality (e.g. neuroticism, extraversion, sensation seeking) in adults (Figure 2B), internalizing problems in children (Figure 2C), externalizing problems in children (Figure 2D) and ADHD in children (Figure 2E); *Brain function and Cognition* including data on EEG (Electroencephalography) power, and cognition assessed with age-appropriate psychometric IQ tests at 5, through 18 years, and educational attainment (Figure 3); *Growth and BMI* including information on Body Mass Index and height across ages (Figure 4A and 4B). Figure 5 summarizes the results for *Metabolic risk factors and Migraine*, including cholesterol, glucose and insulin. The results clearly showed for all traits that MZ correlations (green bars) are higher than DZ correlations (blue bars), showing that genetic factors play a substantial role in nearly all these traits. Analyses of the data showed that for almost all phenotypes, the DZss correlations were equal to the DZos correlations. The DZ twin correlations from the most parsimonious model which constrained DZss and DZos correlations to be similar are also provided in figures 1, 2, 3, 4, 5 (orange bars).

**Figure 6:**
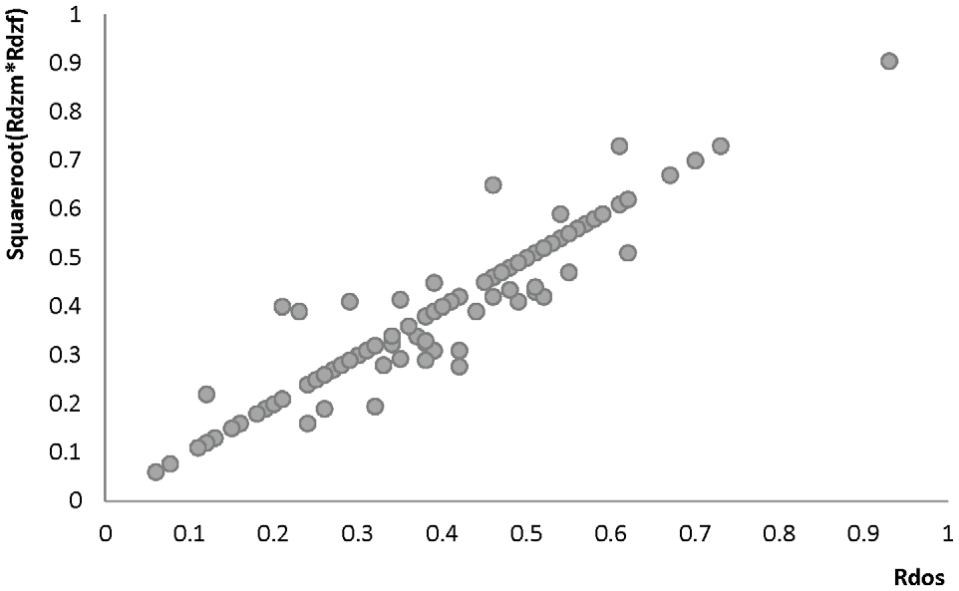
Summary of all observed correlations in opposite-sex twin (x-axis)pairs versus expected correlations correlations based on same-sex dizygotic twin correlations pairs (y-axis) for all 122 phenotypes included in the present paper.

Additionally, a plot of the observed correlation in DZos against its expected value based on the DZss (male-male and female-female) correlations is shown in Figure 6. This figure reflects the similarities and differences between the correlations in same-sex twin pairs and opposite-sex twin pairs. There are few traits for which the resemblance of opposite-sex pairs deviates from the expectation based on the same-sex correlations. For *Lifestyle*, all correlations in adult DZos twins equaled those in DZss twins. In adolescents, sex-differences were observed for Ever Use of Alcohol and Weekly Alcohol Use (see Figure 1A and 1B). Additional tests for these traits suggest that twins from DZos pairs shared fewer environmental factors than twins from same-sex DZ pairs (φ respectively 0.79 and 0.66), see Table S7B. For *Emotional and Behavioral Problems* significantly lower correlations were found in adult DZos twins for Thrill and Adventure Seeking and Sensation Seeking (Figure 2A and 2B). The subsequent model fitting analyses for these traits (Table S7A, B, C) suggested that different genes are expressed in adult men and women (γ in DOS pairs of respectively 0.36 and 0.2). The childhood data showed higher correlations in dos-twin pairs compared to same- sex twin pairs for 24 of the 40 traits (Figure 2C–2E), suggesting that complex mechanisms such as social interactions, or rater contrast effects [8]–[9] may play a role when parental ratings of child behaviors are analyzed. For phenotypes from the *Brain and Cognition* domain, all correlations were equal in DZss and DZos pairs (Figure 3), except for adult Educational Attainment where a higher correlation in DZF twin pairs compared to DZM or DZos twin pairs was seen, suggesting that sex-linked dominant loci could play a role [10]. No sex-differences were observed for *BMI and Height* in adults (Figure 4A). In the younger sample (Figure 4B), there were some differences in DZ twin correlations for BMI in 14-year old twin pairs and DZos correlations for Height were significantly lower in 7-year old twins compared to DZss pairs Figure 5 shows no differences between correlations in DZss or DOS- twin pairs for *Metabolic risk factors and Migraine*, except for HDL-cholesterol.

## Discussion

Evidence for sex-differences in the genetic architecture of complex human traits was found for 6 of the 122 variables (in 4 traits the difference was explained by different genes in men and women and in 2 traits by different environmental influences in men and women). The observed number is in line with that expected if the type-I error rate is 5%, indicating that in our data sex-differences in the genetic architecture of complex phenotypes are rare. Power analyses (see Table S6) indicated that power in our samples is sufficient to detect sex by genotype interactions for nearly all traits. To attain sufficient power to detect differences between DZss and DZos correlations (based on likelihood-ratio tests) sample sizes need to be larger as the correlations between relatives decrease. The smallest samples sizes in this study were for measures of brain function, which tend to show high heritability and high correlations in first-degree relatives, while the low DZ correlations were for traits such as birth weight for which sample sizes were large.

As Eaves and colleagues (1978) pointed out, the comparison of similarity in DZos twin pairs and DZss twin pairs can reveal important information on sex-dependent effects in genetic factors. In 1997, Eaves and colleagues reported they did not find striking evidence that different genetic or environmental factors affect males and females, for a wide range of adolescent behavioral traits like anxiety and depression, conduct disorder, ADHD, impulsivity [11].There are several other examples of twin studies that did not find evidence for sex differences, for example for substance use [12] and depression [13] although some other studies find sex differences, for example for BMI [14] and conduct disorder [15]. A large study of cardiovascular and personality traits in 6,148 Sardinians concluded that despite heterogeneity in effect sizes (in general higher heritability in females compared to males), the same loci appear to contribute to variance in males and females [16].

In the past decennia, the field of genetic research has developed rapidly. Due to advanced measurement techniques, gene finding strategies shifted from candidate genes studies and linkage analyses, to genome-wide association studies and whole genome sequencing. There are few systematic large-scale studies that have addressed sex-differences in the genetic architecture of complex traits or in the genetic associations of such traits with candidate genes or genome-wide SNP data. For anthropometric measures, 7 of the 348 SNPs displayed significant sex difference in large genome-wide search including over 60.000 men and over 70.000 women (Randall et al, under review) [17]. A systematic appraisal of 432 sex-difference claims from 77 genetic association studies concluded that most claims were insufficiently documented or spurious. Claims with documented good internal and external validity were uncommon [18] leading to a recommendation that gene-sex interactions should be based on *a priori*, clearly defined, and adequately powered subgroup analyses, should be explained with caution, and be replicated.

No systematic reviews are available of sex differences in GWA studies. Therefore, we reviewed all available GWA studies for *Height* on sex-specific analyses and/or results. We selected this phenotype because it met criteria proposed in Visscher et al [19]: at least three GWAS papers published in journals with a 2010–2011 journal impact factor >9 and at least one paper containing 10 or more genome-wide significant loci. We considered all GWA studies published until the second quarter of 2011, and found 19 that included the phenotype Height [20]. In eleven studies sex-effects were considered in some way. One study reported sex-heterogeneity at 3 SNPs, and one study reports a larger effect size in females for 1 SNP, while the other studies did not detect sex-specific loci (See Table S8). In conclusion, sex-specific effects were small or absent, with only a few exceptions. This is in line with our observations in the present paper. We did not detect qualitative sex-differences in adult height, and some small differences in the height data of children.

Based on our systematic comparisons of resemblance in DZ twin pairs conditional on sex in a large dataset, we find that sex by genotype interactions for a broad range of behavioral, psychiatric and health related phenotypes are rare. These results suggest that for most variables there is no strong *a priori* hypothesis that different genes are expressed in men and women. This does not imply that it is not worth to search for sex-specific genes in GWAS because there might still be cases of sex-specific effects at specific loci that are overshadowed by polygenic variance.

## Materials and Methods

### Ethics statement

All projects that supplied data have been reviewed and approved by the Central Ethics Committee on Research Involving Human Subjects of the VU University Medical Center, Amsterdam , an Institutional Review Board certified by the US Office of Human Research Protections (IRB number IRB-2991 under Federal wide Assurance-3703; IRB/institute code NTR 03-180). For non-survey research projects involving adults (18 years and above) participants provide written informed consent themselves. For children younger than 12 years, their parents or legal representatives give written informed consent; for children between 12 and 18 years, both parent and the children themselves sign the informed consent forms.

### Study samples

The Netherlands Twin Register (NTR) consists of twins and their families who participate in longitudinal research projects. Young twins (YNTR) are registered at birth by their parents [21]. Demographic characteristics, recruitment and data collection procedures in these samples have described in detail elsewhere [22]. In short, parents were asked to report on their twins by survey at ages 0, 2, 3, 5, 7, 9 and 12 years, and additional teacher reports were collected at age 7, 9 and 12. The twins were asked for self-report information at the ages 14, 16 and 18 years. YNTR twins and siblings are included in the ANTR surveys after age 18. Adolescent and adult twins (ANTR) have been recruited through City Councils in 1990–1993 and through additional efforts such as newsletters and advertisements. ANTR participants (twins and their family members) took part in longitudinal survey studies in 1991, 1993, 1995, 1997, 2000, 2002, 20004/5 and 2009/11. Cross-sectional datasets were created in which the most recent data from each twin pair were selected. A large group of twins participated in the NTR Biobank study, between 2004 to 2008. Details are described elsewhere [23].

The variables are grouped in 5 domains. All variables are described in more detail in Methods S1.

### 1. Lifestyle

#### A. Adults

*Ever smoked; Current smoking, Nicotine dependence, Cannabis use, Regular drinking, Alcohol problem, Exercise participation, Coffee consumption*

#### B. Adolescents

*Ever smoked; Current smoking, Cannabis use, Ever alcohol, Early alcohol initiation at age 13–15, Weekly drinking.*

### 2. Emotional and Behavioral Problems

#### A. Psychiatry adults

*Anxious depression, Anxiety, Social Fear, Blood Fear, OCD (obsessive compulsive disorder) Symptoms, Borderline personality features, Anger, Loneliness, ADHD (Attention Deficit Hyperactivity Disorder)*

#### B. Personality adults

*Extraversion, Neuroticism, Conscientiousness, Agreeableness*

*Openness to Experience, Thrill and Adventure seeking, Disinhibition, Experience Seeking, Boredom Susceptibility, Sensation Seeking.*

#### C. Internalizing behavior Children

*Anxious Depression, Internalizing, Somatic Complaints, Withdrawn Behavior.*

#### D. Externalizing behavior children

*Externalizing, Aggressive behavior, Oppositional defiant problems, Rule-breaking Behavior.*

#### E. Other scales children

*Social problems, Thought problems, Sleep problems, Total problems, Overactive, Attention problems.*

### 3. Brain and Cognition

*F3 power from* **Electroencephalography** *(EEG) recordings, IQ (intelligence quotient), educational attainment.*

### 4. Growth and BMI

*Birth weight, Height, Body Mass Index (BMI).*

### 5. Metabolic risk factors and migraine

*Blood pressure (BP), fasting Total cholesterol*, **High-density lipoprotein** (*HDL*), **Low-density lipoprotein** *(LDL), triglycerides, fasting glucose, fasting insulin and HbA1C, Fibrinogen, C-reactive protein (CRP),Tumor necrosis factor-alpha (TNF-α), Interleukin-6 (IL-6), Interleukin-receptor-6 (IL6R)*, A*spartate Aminotransferase (AST)*, A*lanine Aminotransferase (ALT)*, G*amma-Glutamyl-Transferase (GGT)*, *Migraine*.

The variables are described in more detail in Methods S1.

### Power analyses

Power analyses were run to explore the sample size necessary to detect a significant difference between the correlation in DZ same-sex (DZss) twin pairs and DZ opposite-sex (DZos) twin pairs. Using an MX-script [24], we tested the difference between DZss and DZos correlations by likelihood-ratio tests. The required sample sizes for statistical power ranging from .75 to .99 (with significance level 0.05 and 1 degree of freedom) are shown in Table S6.

### Statistical analyses

Statistical analyses were performed with genetic structural equation modeling as implemented in the software package Mx [24]. For continuous variables, a so-called saturated model was fitted to the data in which means (for men and women), variances (for men and women) and five twin correlations were estimated. The regression of age (z-value) or year of birth was (z-value) (separately for man and women) was modeled as a fixed effect, allowing for a linear decrease or increase of the mean with age or cohort. For the dichotomous variables, a threshold model was applied, in which a trait is assumed to have an underlying continuous liability with a standard normal distribution with zero mean and unit variance. Thresholds divide this normal distribution into discrete categories [3]. Different thresholds were estimated for men and women. A regression of the z-score of age or cohort was modeled as a fixed effect on the threshold.

With this saturated model (model 1) as a baseline model, a series of models was evaluated:

In model 1a the variances were constrained to be equal (for continuous data only). In model 2 the correlation in DZM pairs was constrained to the correlation in DFZ pairs (rdzm = rdzf) while in model 3 those correlations were also constrained to the correlation in dizygotic opposite-sex twin pairs (rdzm = rdzf = rdos). Testing of models was done by likelihood-ratio tests, by subtracting the negative log-likelihood (−2LL) for the more restricted model from the −2LL for the more general model. This yields a statistic that is distributed as chi square with degrees of freedom (df) equal to the difference in the number of parameters in the two models. If the difference test is significant (p<0.05) the constraints on the nested model cause a significant deterioration of the model.

When the DZos correlation was significantly lower than the DZss correlation, an additional set of analyses was carried out to estimate variance components due to Additive genetic effects (A), Common environmental effects (C) and unique Environmental effects (E). The analysis of twin data rests critically on several assumptions. One is that the environmental components of variance are the same in the two types of twins (MZ versus DZ) and another one that the total genetic variance is the same in the two types [1]. These and other assumptions are addressed in more detail in van Dongen et al [25]. In general, the empirical evidence suggests that these assumptions are reasonable.

For the traits that showed sex differences in the saturated model, additional model fitting was carried out. First a full model that allowed the magnitude of A, C and E to be different in men and women was fitted to the data. In this model, the genetic correlation in DZos twins (γ) was allowed to be smaller than 0.5 (or if the C component was much larger than the A component, the shared environmental correlation in DOS twins (φ) was allowed to be smaller than 1). It should be noted that there is a particular problem in trying to differentiate between γ and φ, these are confounded and can only be estimated by making some very strong assumptions.

In the next model all variance components were constrained to be the same in men and women. We tested whether variance components due to A and C were significantly different from zero. Finally, γ, the genetic correlation in DOS twins, was constrained at 0.5 (or φ , the shared environmental correlation, was fixed to 1). Significance of the parameters was tested by comparing the fit of the nested models to the fit of less restricted models. Goodness-of-fit of the sub models was assessed by likelihood-ratio test. The difference in log-likelihoods between the nested models follow a χ2 distribution, with degrees of freedom (df) equal to the diference in the number of paramaters in the two models. According to the principle of parsimony, models with fewer parameters are preferred if they do not give a significant deterioration of the fit (p>0.01).

## Supporting Information

Methods S1 Comprehensive description of the variables and measures.
(DOC)

Table S1 Lifestyle. (A) Lifestyle Adults. (B) Lifestyle Adolescents
(DOC)

Table S2 Emotional and Behavioral problems. (A) Emotional and Behavioral problems - Psychiatry adults (B) Emotional and Behavioral problems - Personality adults. (C) Emotional and Behavioral problems – Internalizing children (D) Emotional and Behavioral problems – Externalizing children (E) Emotional and Behavioral problems – others children
(DOC)

Table S3 Brain and Cognition
(DOC)

Table S4 BMI and Height. (A) BMI and Height – adult (B) BMI and Height – children and adolescents
(DOC)

Table S5 Cardiovascular, metabolic and migraine
(DOC)

Table S6 Number of twin pairs required to detect significant difference between correlation in DZ same sex (DZss) twin pairs and DZ opposite sex (DZos) twin pairs.
(DOC)

Table S7 Significantly different DZ correlations (A) Twin correlations for traits with significantly lower DZ opposite-sex correlations than DZ same-sex correlations. For these traits, a genetic model was fitted to the data to test whether the difference was due to different genes being expressed in men and women or environmental factors being less correlated in opposite-sex pairs (see Supplementary Table 11C and D for results). (B) Full model including additive genetic, common and unique environmental factors (a^2^, c^2^, and e^2^ give explained variance for traits with evidence for qualitative sex differences; γ and φ represent respectively the genetic correlation and environmental correlation in DZ-opposite sex twin pairs (C) Parameter estimates based on most parsimonious model (D) Overview of the twin correlations for traits where DZ correlations were significantly different from each other (but Rdos not lower than Rdzm/Rdzf). No additional models were fitted.
(DOC)

Table S8 Overview of all published GWA studies for Height based on the database published on www.genome.gov/gwastudies (February 2011) and literature search in pub med. We selected this phenotypes because it is representative selection among all complex traits and it meets the criteria proposed by Visscher et al: at least three GWAS papers published in journals with a 2010–2011 journal impact factor >9 and at least one paper containing 10 or more genome-wide significant loci. We found 19 GWA studies and examined whether the studies considered sex differences, and if yes, whether they found significant sex differences.
(DOC)
