# Supplementary material for: Sex Differences in Genetic Architecture of Complex Phenotypes?
Source: PLoS One. 2012 Dec 18;7(12):e47371. doi: 10.1371/journal.pone.0047371 (PMC3525575; doi:10.1371/journal.pone.0047371)
Supplement: Methods S1 — Comprehensive description of the variables and measures. (DOC) [file pone.0047371.s001.doc]

Supplementary Methods S1

S**ex differences in genetic architecture of complex phenotypes**

**Methods section**

***1. Lifestyle***

-*Ever smoked:* Both ANTR and YNTR subjects were classified as never smokers when they reported they never smoked or when they tried smoking a few times but never smoked regularly [1].

-*Current smoking:* The answer categories to the question “How often do you smoke now?” were collapsed into current smoking yes (I smoke once a week or less, I smoke a few times a week, not every day, I smoke once or several times a day) and current smoking no (I’ve never been a regular smoker, I’ve quit smoking) both for ANTR and YNTR cohorts [2].

-*Nicotine dependence:* The Fagerstrom Test for Nicotine Dependence consist of 6 items and produces a score ranging from 0 to 10 with higher scores indicating more nicotine dependence [3-4]. The FTND score (among ANTR smokers and ex-smokers) was collapsed into a dichotomous variable (FTND >= 4 versus FTND < 4).

- *Cannabis use:* In the ANTR, subjects were asked at what age they initiated cannabis use and the answers were re-coded in the variable ‘cannabis initiation’, with two possible categories; ‘yes’ when a subject initiated cannabis use (irrespective of age), or ‘no’ when a subjected never initiated cannabis use [5]. In the YNTR the Dutch Health Behavior Questionnaire (DHBQ) was included. Subjects were asked whether they ever used cannabis and, if so, how many times. Responses were re-coded into the variable ‘initiated cannabis’ with two possible categories: 0, when a subject never used cannabis; and 1, when a subject had ever used cannabis [6].

-*Ever alcohol* in YNTR: Have you ever used alcohol was re-coded to never (never and 1or 2 times) versus ever (3 times or more).

-*Regular drinking* in ANTR: Regular drinking defined as: >= 21 glasses per week for men and >= 15 glasses per week for women (conform Nationaal Kompas Volksgezondheid [www.nationaalkompas.nl](http://www.nationaalkompas.nl/) (dutch)).

-*Weekly drinking* YNTR: “How often do you drink an alcohol beverage”: answer categories were collapsed into: once a week or more often (answer 5,6,7) versus a few times per month or less (answer 0,1,2,3,4).

-*Alcohol problems* in ANTR : Alcohol problems were assessed by CAGE. The CAGE, originally developed as a screening instrument in medical settings [7], is a short questionnaire to obtain an indication of the presence of Symptoms of AAD [8-9]. Its four items are: Have you ever felt that you should Cut down your drinking?; Have people Annoyed you by criticizing your drinking?; Have you ever felt bad or Guilty about your drinking?; Have you ever had a drink in the morning (as an ‘Eye opener’) to steady your nerves or get rid of a hangover? The items can be answered with ‘yes’ or ‘no’. In 2009/2010, there were two yes categories: ‘yes, during the last year’; ‘yes, not during the last year’ that, for these analyses, were re-coded into one, since its combined frequency was comparable to the frequency of the single yes categories in earlier years. If one of the four CAGE items was missing (N = 296), the missing value was imputed based on the mean of the three other answers. For individuals with two or more missing items (N = 187) and those who reported to never have drunk any alcohol (N=332), the CAGE was set to missing. For the current study, we analyzed the number of ‘yes’ items as 0, 1, 2 and 3+, combining the 3 and 4 yes positive answers as as the number of individuals who answered four items positively, was low (N=56).

-*Early alcohol initiation at age 13-15* (in YNTR sample) was defined as ever having used alcohol, to which response categories were ‘no’, ‘a few times’ and ‘yes’. The categories ‘a few times’ and ‘yes’ were collapsed, resulting in a binary variable [10].

-*Coffee:* The ANTR survey of 2000 contained the question: ‘On average, how many cups of caffeinated coffee do you drink in one day?’ A large part of the subjects reported drinking no coffee at all causing a skewed distribution. Therefore, the data were divided in categories: zero to two cups per day, three to five cups per day and six or more cups per day [11].

-*Exercise participation* in ANTR was measured with a number of questions. The first question was ‘Do you participate in exercise regularly?’. This question could be answered with Yes or No. If the participants responded affirmative, further information on type, frequency and duration of exercise was gathered. All exercise activities were assigned a metabolic equivalent value, using Ainsworth’s Compendium of physical activity [12]. A metabolic equivalent score of 1 corresponds to the rate of energy expenditure when at rest (1 kcal/kg/h).

Exercise participation was defined as a dichotomous variable, classifying participants as either regular exerciser or non-exerciser [13]. A cutoff criterion of exercising at four metabolic equivalents or more for at least 60 minutes a week in the recent year was used to classify participants as regular exercisers. Exercise participation was assessed in ANTR surveys 1991, 1993, 1995, 1997, 2000, 2002 and 2004. For each twin pair, valid exercise data from the most recent survey were selected.

***2. Emotional and Behavioral Problems***

***Obtained from longitudinal surveys in adults (ANTR, 18-65 years)***

-*NEO personality scales:* Personality scores for the five factors Neuroticism, Extraversion, Openness to Experience, Agreeableness and Conscientiousness were assessed with the 60 items of the NEO-FFI (12 items per factor) [14-15]. Items were answered on a 5-point Likert-type scale ranging from strongly disagree (1) to strongly agree (5). Summed scores were computed for all five personality dimensions (after reversing negatively keyed items). If more than 10 items were missing, summed scores were not computed. If less than 10 items were missing, the missing items were given the neutral option (2). Sumscores for each dimension could range from 12 to 60. Personality data came from ANTR survey 7 (2004).

-*Fear* in ANTR: Blood-injury fear (e.g., avoidance of hospitals) and Social fear (e.g., avoiding talking to people in authority) were measured by the Dutch version of the Fear Questionnaire (FQ) [16]. Both domains are represented with 5 items. Subjects were instructed to indicate on a nine point scale (0 ‘would not avoid it’ to 8 ‘always avoid it’) how much they would avoid certain situations because of fear or other unpleasant feelings. Scores were summed across subscales. A score was only calculated if at least 4 out of 5 items of a subscale were answered. Missing answers and double entries were substituted by the mean item score. The sum scores were transformed to a dichotomous measure.

-*Obsessive compulsive symptoms* (OCS): OC symptoms were measured by 12 items of the Padua Inventory [17], translated into Dutch, revised and validated by Van Oppen et al. [18]. Items were chosen from each OC subscale of the Padua Inventory Revised. The sensitivity and specificity for the 12 items to detect OCD were 0.74 and 0.72 respectively, when comparing a group of OCD patients with clinical controls [19].

- *Borderline personality features* were measured by the Dutch translation of the Personality Assessment Inventory-Borderline Features scale (PAI-BOR) [20-21]. PAI-BOR items tap features of severe personality pathology that are clinically associated with borderline personality disorder and consists of 24 items that are rated on a four-point scale (0 to 3; false, slightly true, mainly true, very true). The PAI-BOR was scored according to Morey’s test manual [20], which states that at least 80% of the items must be answered to calculate a sum score and that missing and ambiguous answers should be substituted by a zero score.

-*Anxious depression* –Anxious depression was measured with the Young Adult Self Report (YASR) [22-23].

-*State-Trait Anxiety:* Anxiety was measured with the Dutch translation of the Spielberger State Trait Anxiety Inventory – Trait version (STAI) [24-25].

- *State-Trait Anger*: Trait anger was measured with the Dutch adaptation of the State Trait Anger Scale (STAS) [26-27]. The scale is designed to assess the frequency of which an individual experiences the state anger over time and in response to a variety of situations. The 10-item STAS-adaptation was scored on a 4-point likert scale (1-4; almost never, sometimes, often, almost always). Participants were asked to indicate the extent to which an item occurred in their everyday lives. The items were scored according to the test manual, which states that at least 80% of the items must be answered to calculate a sum score and that missing values or ambiguous answers should be substituted by a score of 2 (sometimes true).

-*Loneliness* was assessed with the short scale for measuring loneliness in large surveys, developed by Hughes et al.[28]. The three items that compose this scale were

selected from the R-UCLA Loneliness Scale [29]: ‘‘How often do you feel left out’’, ‘‘How often do you feel isolated from others’’, and ‘‘How often do you feel that you lack companionship’’. Response categories were: ‘‘Hardly ever’’ (1), ‘‘Some of the time’’ (2), and ‘‘Often’’(3). The data were analysed as an ordinal measure with 4 categories.

-*Sensation seeking* was measured with the Dutch translation of the Sensation Seeking Scale (SSS) from Zuckerman [30-32]. The sensation seeking scale contained 51 items that measured four dimensions of sensation seeking. The dimension ‘Thrill and adventure seeking’ contained 12 items, ‘Experience seeking’ had 14 items, ‘Boredom susceptibility’ contained 13 items and ‘Disinhibition’ was assessed with 12 items . Items were answered on a 5-point Likert-type scale ranging from totally disagree (1) to totally agree (5). Summed scores were computed for all four dimensions (after reversing negatively keyed items). If more than 2 items were missing per dimension, summed scores were not computed. If less than 2 items were missing per dimension, missingness was imputed with the average value on that dimension for a particular individual. In addition, an overall sensation seeking score was computed by adding the summed scores of the four dimensions divided by the number of items (sss = tas/12 + es/14 + bs/13 + dis/12). Data on sensation seeking came from ANTR surveys 1991, 1993, 1997, 2000 and 2002.

***Behavioral and emotional problems in children (YNTR, 3-12 years)***

Parental ratings of behavioral and emotional problems in 3 to 12-year old children were collected by age appropriate versions of the Child Behavior Checklist, which are part of the Achenbach System of Empiral Based Assesment (www.aseba.org). Details of data collection are described elsewhere [33-35]. Parents report on the occurrence of behavioral problems in their children on a 3-point scale: 0 if the problem item was not true, 1 if the item was somewhat or sometimes true, and 2 if it was very true or often true. A syndrome scale is a summation of the corresponding items. At age 3, seven syndrome scales are obtained *(Emotionally Reactive, Anxious/Depressed, Somatic Complaints, Withdrawn, Overactive Behavior, Aggressive Behavior, Sleep)*, 2 broad groupings of syndromes (*Internalizing and Externalizing Problems)*, and the Total Problem score. At ages 7, 10, and 12 eight syndrome scales are obtained (*Anxious/ Depressed, Withdrawn, Somatic Complaints, Social Problems, Thought Problems, Attention Problems, Rule-Breaking Behavior)* and 2 broad problems scales are obtained *(Internalizing and Externalizing Problems).*

***3. Brain and Cognition***

*EEG* was obtained in twins aged 5 through 71 years during 3–4 min of eyes-closed rest. After visual and semi-automated data cleaning, including removal of independent components linked to blink and heartbeat artifacts, EEG power was determined for the left frontal F3 electrode using Welch’s periodogram method implemented in MATLAB. A full description of the sample and EEG registration/preprocessing can be found elsewhere (Smit et al., 2011).

*Cognition* data were obtained with age-appropriate psychometric IQ tests: RAKIT at ages 5, 7, and 10, the RAKIT, WISC at age 12, and WAIS at age 18. Data were collected in lab studies by trained research-assistants. Educational level (CITO scores) was assessed by standardized tests in 12-year olds during a 3 day testing period and these data were obtained from the parents and teachers of twins [36].

-*Educational attainment* (EA). In all ANTR surveys, participants were asked to indicate their highest educational level and whether or not they had completed this education with a diploma [37]. To obtain one measure of educational attainment for each participant, all adult data (age 21 years or older) for an individual were combined into a measure with 4 categories:

1. primary school; 2. lower vocational education and general secondary education; 3. intermediate vocational education and higher secondary education; 4. higher vocational education (HBO) and college/university education.

***4. Growth and BMI***

*Birth weight*: In YNTR, parents of twins reported birth weight in survey-1, which is collected after parents register their twins (age < 1 years) [38]. In the ANTR cohort, participants provided self-report birth weight in most surveys. In addition, parents reported on the birth weights of their offspring. All data available for an individual were combined and checked for consistency across time and rater. Birth weight was determined as the average of all valid data points, when the discrepancy was no larger than 200 grams [39].

*Height:* All ANTR surveys asked participants to provide their body height in cm. Data provided for the age of 18 years and above were checked for consistency across time. When the difference across time points did not exceed 5 cm, the height data were averaged to come to one adult body height [40]. In YNTR-surveys for 2 and 3 year olds), a parent was asked to report height and weight as measured by the routine health care program in the Netherlands (Youth Health Services). In follow-up surveys (ages 5, 7, 10, 12 years) a parent was asked to report current weight and height. From age 14 onwards, the height and weights were self-reported by the twin.

*-BMI*: Body mass index (BMI) was obtained from maternal report in children and from self-report in adolescents and adults. BMI was calculated as weight_(kg)_ / height^2^_(m)_. We used the body height measure as described above and weight from the most recent study in which both twins participated, or if this never was the case, the most recent study [41-42].

***5. Metabolic risk factors and migraine***

The metabolic risk factors were measured in blood samples of twins who participated in the NTR biobank study [43].

- *Blood pressure* (BP) data were collected in multiple studies. BP was measured using brachial cuff measurements on the non-dominant arm by an automated Dynamap 845 recorder in the laboratory [44] or by the Spacelabs 90207 ambulatory monitoring device [45]. The laboratory measurements were obtained with subjects sitting quietly for periods varying from 3 to 8 minutes. A mean of 3 to 6 BP measurements was taken. In the ambulatory BP study the mean of all evening measurements when subjects were seated calmly was taken as the resting value (mean number of measurements 3.8,). The BP values in all four studies were corrected for the use of antihypertensive medication [46-47]. If subjects were taking antihypertensive medication a correction of +14 mmHg for SBP and +10 mmHg for DBP was made for the laboratory studies. For the ambulatory study, the correction was drug class specific, but average values were close to +14 mmHg for SBP and +10 mmHg for DBP.

- *Lipids* (total cholesterol, HDL, LDL and triglycerides) were assessed after overnight fasting [43]. Total cholesterol HDL-cholesterol and triglyceride levels were measured in heparin plasma using the Vitros 250 total cholesterol assay, the Vitros 250 direct HDL cholesterol assay and the Vitros 250 Triglycerides assay (Johnson & Johnson, Rochester, USA). LDL-cholesterol was calculated using the Friedewald formula [48]. Subjects using lipid lowering medication at the time of blood sampling were excluded from the analyses. For HDL, one extreme outlier was excluded from the analysis (HDL=6.56). For triglycerides an LN-transformation was applied .

-*glucose metabolism* (fasting glucose, fasting insulin and HbA1C) parameters were assessed after overnight fasting [43]). Blood glucose was measured using the Vitros 250 Glucose assay (Johnson & Johnson, Rochester, USA) and insulin was measured using the Immulite 1000 Insulin Method (Diagnostic Product Corporation, Los Angeles, USA). Hba1c was determined in EDTA whole blood using the Nyocard HbA1c assay (Axis-Shield, Oslo, Norway). For the analyses of glucose, insuline and all lipids, subjects who had not fasted from 24h the night before the home visit were excluded, and subjects taking medication for diabetes and subjects with a fasting glucose level >= 7 were excluded. For insuline an LN-transformation was applied .

*-Fibrinogen* was measured in a 4.5 ml CTAD tube that was stored during transport in melting ice. Fibrinogen levels were determined on a STA Compact Analyzer  Diagnostica Stago, France), using STA Fibrinogen (Diagnostica Stago, France). For fibrinogen a cut off value of 5 pg/ml was used for exlusion of subjects.

*-C-reactive protein (CRP)* was measured in heparin plasma, CRP level was determined using the Immulite 1000 CRP assay (Diagnostic Product Corporation, USA). Data were excluded when values of CRP rose above 25 pg/ml,

*-Tumor necrosis factor-alpha (TNF-α), Interleukin-6 (IL-6)* and *Interleukin-receptor-6 (IL6R)* were measured in EDTA plasma, using R&D systems. IL6 and TNF- α data were excluded when values exceeded 15pg/ml and IL6-R data were excluded when exceeding 100.000 pg/ml.

-Data on the liver enzymes A*spartate Aminotransferase (AST)*, A*lanine Aminotransferase (ALT)* and G*amma-Glutamyl-Transferase (GGT)* were determined in plasma that was collected in heparin plasma tubes using Vitros assays were used (Willemsen et al., 2010). Liver enzyme levels were excluded for six individuals with liver disease (ICD-10 codes K70-K77), 12 individuals with extreme outliers (AST >79 U/L; ALT >63 U/L; GGT >296 U/L; values >9 SD above the mean) and four twins without information on zygosity, resulting in a final data set of 3,662 individuals. To obtain more normally distributed variables, liver enzyme levels were ln-transformed and multiplied by 10 to avoid problems during estimation that result when analyzing very small covariances.

-*Migraine* was assessed with ANTR survey items that cover the symptoms included in the official diagnostic criteria for migraine (ICHD-II) as defined by the International Headache Society. The item variables were analyzed with latent class analysis [49], resulting in four groups of individuals, empirically classified based on their patterns of headache symptomatology, which differed primarily in the severity of their headaches. The two most severely affected classes, (“moderate/severe migraine” and “mild migrainous headache”) were classified as affected. [50].

**References**

1. Vink, J.M., G. Willemsen, and D.I. Boomsma, *Heritability of smoking initiation and nicotine dependence.* Behavior Genetics, 2005. **35**(4): p. 397-406.

2. Vink, J.M., A.B. Smit, E.J.C. De Geus, P. Sullivan, G. Willemsen, J.J. Hottenga, J. Smit, W.J. Hoogendijk, F.G. Zitman, L. Peltonen, J. Kaprio, N.L. Pedersen, P. Magnusson, T.D. Spector, K.O. Kyvik, K.I. Morley, A.C. Heath, N.G. Martin, R.G.J. Westendorp, P.E. Slagboom, H. Tiemeier, A. Hofman, A. Uitterlinden, Y. Aulchenko, N. Amin, C. van Duijn, B.W. Penninx, and D.I. Boomsma, *Genome-wide association study of smoking initiation and current smoking.* The American Journal of Human Genetics, 2009. **84**: p. 1-13.

3. Heatherton, T.F., L.T. Kozlowski, R.C. Frecker, and K.O. Fagerstrom, *The Fagerstrom Test for Nicotine Dependence: a revision of the Fagerstrom Tolerance Questionnaire.* British journal of addiction, 1991. **86**: p. 1119-1127.

4. Vink, J.M., G. Willemsen, A.L. Beem, and D.I. Boomsma, *The Fagerstrom Test for Nicotine Dependence in a Dutch sample of daily smokers and ex-smokers.* Addictive Behaviors, 2005. **30**(3): p. 575-579.

5. Vink, J.M., L. Wolters, M.C. Neale, and D.I. Boomsma, *Heritability of cannabis initiation in Dutch adult twins.* Addictive Behaviors, 2010. **35**(2): p. 172-174.

6. Distel, M.A., J.M. Vink, M. Bartels, C.E.M. van Beijsterveldt, M.C. Neale, and D.I. Boomsma, *Age moderates non-genetic influences on the initiation of cannabis use: a twin-sibling study in Dutch adolescents.* Addiction, 2011. **106**: p. 1658–1666.

7. Ewing, J.A., *Detecting alcoholism—the cage questionnaire.* J Am Med Assoc, 1984. **252**: p. 1905-1907.

8. Dhalla, S. and J.A. Kopec, *The CAGE questionnaire for alcohol misuse: a review of reliability and validity studies.* Clin Investig Med, 2007. **30**(33-41).

9. van Beek, J.H.D.A., K.S. Kendler, M.H.M. De Moor, L.M. Geels, M. Bartels, V. J.M., S.M. van den Berg, G. Willemsen, and D.I. Boomsma, *Stable Genetic Effects on Symptoms of Alcohol Abuse and Dependence from Adolescence into Early Adulthood.* Behavior Genetics, in press.

10. Geels, L.M., M. Bartels, C.E.M. van Beijsterveldt, G. Willemsen, N. van der Aa, J.M. Vink, and D.I. Boomsma, *Trends in adolescent alcohol use: Effects of age, sex and cohort.* Addiction, in press.

11. Vink, J.M., A.S. Staphorsius, and D.I. Boomsma, *A genetic analysis of coffee consumption in a sample of Dutch twins.* Twin Res Hum Genet., 2009. **12**(2): p. 127-31.

12. Ainsworth, B.E., W.L. Haskell, M.C. Whitt, M.L. Irwin, A.M. Swartz, S.J. Strath, W.L. O'Brien, D.R.J. Bassett, K.H. Schmitz, P.O. Emplaincourt, D.R.J. Jacobs, and A.S. Leon, *Compendium of physical activities: an update of activity codes and MET intensities.* Med Sci Sports Exerc., 2000. **32**(9 Suppl): p. S498-504.

13. Stubbe, J.H., D.I. Boomsma, J.M. Vink, B.K. Cornes, N.G. Martin, A. Skytthe, K.O. Kyvik, R.J. Rose, U.M. Kujala, J. Kaprio, J.R. Harris, N.L. Pedersen, J. Hunkin, T.D. Spector, and E.J.C. de Geus, *Genetic Influences on exercise participation in 37.051 twin pairs from seven countries.* PLoS ONE, 2006. **1**(2): p. e22.

14. Costa, P.T. and R.R. McCrae, *Professional manual: Revised NEO Personality Inventory (NEO-PI-R) and NEO Five-factor-Inventory (NEO-FFI)*. 1992, Odessa, FL: Psychological Assesment Resources.

15. Hoekstra, H.A., J. Ormel, and F. De Fruyt, *Handleiding NEO persoonlijkheids-vragenlijsten NEO-PI-R en NEO-FFI.* , ed. S.T. Services. 1996, Lisse.

16. Marks, I.M. and A.M. Mathews, *Brief Standard Self-Rating for Phobic Patients.* Behav Res Ther 1979. **17**(263-267).

17. Sanavio, E., *Obsessions and compulsions: the Padua Inventory.* Behavior Research and Therapy, 1988. **26**(2): p. 169-177.

18. van Oppen, P., R.J. Hoekstra, and P.M. Emmelkamp, *The structure of obsessive-compulsive symptoms. .* Behavior Research and Therapy, 1995. **33**: p. 15-23.

19. Cath, D., D. van Grootheest, G. Willemsen, P. van Oppen, and D. Boomsma, *Environmental Factors in Obsessive-Compulsive Behavior: Evidence from Discordant and Concordant Monozygotic Twins.* Behavior Genetics, 2008. **38**(2): p. 108-120.

20. Morey, L.C., *The Personality Assessment Inventory: Professional manual.* 1991, Odessa, FL: Psychological Assessment Resources.

21. Distel, M.A., M.H.M. de Moor, and D.I. Boomsma, *Nederlandse vertaling van de Personality Assessment Inventory- borderline schaal (PAI-BOR): Normgegevens, factorstructuur en betrouwbaarheid.* Psychologie en Gezondheid, 2009. **37**(38-46).

22. Achenbach, T.M., *The Young Adult Self Report and Young Adult Behavior Checklist.* 1990, Burlington, VT: VT: University of Vermont, Department of Psychiatry.

23. Verhulst, F.C., J.v.d. Ende, and H.M. Koot, *Handleiding voor de Youth Self-Report*. 1997, Afdeling Kinder- en Jeugdpsychiatrie, Sophia Kinderziekenhuis / Academisch Ziekenhuis Rotterdam / Erasmus Universiteit Rotterdam: Rotterdam.

24. Spielberger, C.D., R.I. Gorsuch, and R.E. Lushene, *STAI Manual for the State-Trait Anxiety inventory.*, ed. P. Alto. 1970: CA: Consulting Psychologists Press.

25. Van der Ploeg, H., P.B. Defares, and C.D. Spielberger, *Zelfbeoordelingsvragenlijst STAI, versie DY-1 en DY-2*. 1979, Lisse: Swets & Zeitlinger.

26. Spielberger, C.D., G. Jacobs, J.S. Russell, and R.S. Crane, *Assessment of anger: the state-trait anger scale.* , in *Advances in Personality Assessment. Vol. 2.*, J.N.B.C.D. Spielberger, Editor. 1983, Erlbaum: Hillside, NJ.

27. van der Ploeg, H.M., P.B. Defares, and C.D. Spielberger, *Handleiding bij de zelf-analyse vragenlijst ZAV. Een vragenlijst voor het meten van boosheid en woede, als toestand en als episode. Een Nederlandse bewerking van de Spielberger State-Trait Anger Scale.* 1982, Lisse, the Netherlands: Swets & Zeitlinger.

28. Hughes, M.E., L.J. Waite, L.C. Hawkley, and J.T. Cacioppo, *A short scale for measuring loneliness in large surveys—results from two population-based studies.* Res Aging 2004. **26**: p. 655-672.

29. Russell, D., L.A. Peplau, and C.E. Cutrona, *The Revised Ucla Loneliness Scale—concurrent and discriminant validity evidence.* J Pers Soc Psychol, 1980. **39**: p. 472-480.

30. Zuckerman, M., *Dimensions of sensation seeking.* J Consult Clin Psychol, 1971. **36**: p. 45-52.

31. Feij, J.A., P.H. Dekker, J.R. Koopmans, and D.I. Boomsma, *Nieuwe normen en stabiliteitsgegevens voor de Spanningsbehoeftelijst (SBL).* Nederlands Tijdschrift voor de Psychologie, 1997. **52**: p. 131-134.

32. Feij, J.A. and R.W. Van Zuilen, *Handleiding bij de Spanningsbehoeftelijst (SBL)*. 1984, Lisse: Swets & Zeitlinger.

33. Bartels, M., C.E.M. Van Beijsterveldt, E.M. Derks, T.M. Stroet, T.J.C. Polderman, J.J. Hudziak, and D.I. Boomsma, *Young Netherlands Twin Register (Y-NTR): a longitudinal multiple informant study of problem behavior.* Twin Res Hum Genet., 2007. **10**(1): p. 3-11.

34. Bartels, M., E.J.C.G. Van den Oord, J.J. Hudziak, M.J.H. Rietveld, C.E.M. van Beijsterveldt, and D.I. Boomsma, *Genetic and environmental mechanisms underlying stability and change in problem behaviors at age 3, 7, 10 and 12.* Developmental Psychology, 2004. **40**(5): p. 852-867.

35. Derks, E.M., J.J. Hudziak, C.E.M. Beijsterveldt, C.V. Dolan, and D.I. Boomsma, *A Study of Genetic and Environmental Influences on Maternal and Paternal CBCL Syndrome Scores in a Large Sample of 3-Year-Old Dutch Twins.* Behavior Genetics, 2004. **34**(6): p. 571-583.

36. Bartels, M., M.J. Rietveld, G.C. Van Baal, and D.I. Boomsma, *Heritability of educational achievement in 12-year-olds and the overlap with cognitive ability.* Twin Res Hum Genet., 2002. **5**(6): p. 544-553.

37. Stronks, K., H. van de Mheen, J. van den Bos, and J. Mackenbach, *The interrelationship between income, health and employment status.* Int J Epidemiol., 1997. **26**(592-600).

38. Gielen, M., C.E.M. van Beijsterveldt, C. Derom, R. Vlietinck, J.G. Nijhuis, M.P.A. Zeegers, and D.I. Boomsma, *Secular trends in gestational age and birthweight in twins.* Human Reproduction, 2010. **25**(9): p. 2346-2353.

39. Freathy, R.M., D.O. Mook-Kanamori, U. Sovio, I. Prokopenko, N.J. Timpson, D.J. Berry, N.M. Warrington, E. Widen, J.J. Hottenga, M. Kaakinen, L.A. Lange, J.P. Bradfield, M. Kerkhof, J.A. Marsh, R. Magi, C.-M. Chen, H.N. Lyon, M. Kirin, L.S. Adair, Y.S. Aulchenko, A.J. Bennett, J.B. Borja, N. Bouatia-Naji, P. Charoen, L.J.M. Coin, D.L. Cousminer, E.J.C. de Geus, P. Deloukas, P. Elliott, D.M. Evans, P. Froguel, B. Glaser, C.J. Groves, A.-L. Hartikainen, N. Hassanali, J.N. Hirschhorn, A. Hofman, J.M.P. Holly, E. Hypponen, S. Kanoni, B.A. Knight, J. Laitinen, C.M. Lindgren, W.L. McArdle, P.F. O'Reilly, C.E. Pennell, D.S. Postma, A. Pouta, A. Ramasamy, N.W. Rayner, S.M. Ring, F. Rivadeneira, B.M. Shields, D.P. Strachan, I. Surakka, A. Taanila, C. Tiesler, A.G. Uitterlinden, C.M. van Duijn, A.H. Wijga, G. Willemsen, H. Zhang, J. Zhao, J.F. Wilson, E.A.P. Steegers, A.T. Hattersley, J.G. Eriksson, L. Peltonen, K.L. Mohlke, S.F.A. Grant, H. Hakonarson, G.H. Koppelman, G.V. Dedoussis, J. Heinrich, M.W. Gillman, L.J. Palmer, T.M. Frayling, D.I. Boomsma, G.D. Smith, C. Power, V.W.V. Jaddoe, and M.-R. Jarvelin, *Variants in ADCY5 and near CCNL1 are associated with fetal growth and birth weight.* Nat Genet, 2010. **42**(5): p. 430-435.

40. Lango Allen, H., K. Estrada, G. Lettre, S.I. Berndt, M.N. Weedon, F. Rivadeneira, C.J. Willer, A.U. Jackson, S. Vedantam, S. Raychaudhuri, T. Ferreira, A.R. Wood, R.J. Weyant, A.V. Segre, E.K. Speliotes, E. Wheeler, N. Soranzo, J.-H. Park, J. Yang, D. Gudbjartsson, N.L. Heard-Costa, J.C. Randall, L. Qi, A. Vernon Smith, R. Magi, T. Pastinen, L. Liang, I.M. Heid, J.a. Luan, G. Thorleifsson, T.W. Winkler, M.E. Goddard, K. Sin Lo, C. Palmer, T. Workalemahu, Y.S. Aulchenko, A. Johansson, M. Carola Zillikens, M.F. Feitosa, T. Esko, T. Johnson, S. Ketkar, P. Kraft, M. Mangino, I. Prokopenko, D. Absher, E. Albrecht, F. Ernst, N.L. Glazer, C. Hayward, J.-J. Hottenga, K.B. Jacobs, J.W. Knowles, Z. Kutalik, K.L. Monda, O. Polasek, M. Preuss, N.W. Rayner, N.R. Robertson, V. Steinthorsdottir, J.P. Tyrer, B.F. Voight, F. Wiklund, J. Xu, J. Hua Zhao, D.R. Nyholt, N. Pellikka, M. Perola, J.R.B. Perry, I. Surakka, M.-L. Tammesoo, E.L. Altmaier, N. Amin, T. Aspelund, T. Bhangale, G. Boucher, D.I. Chasman, C. Chen, L. Coin, M.N. Cooper, A.L. Dixon, Q. Gibson, E. Grundberg, K. Hao, M. Juhani Junttila, L.M. Kaplan, J. Kettunen, I.R. Konig, T. Kwan, R.W. Lawrence, D.F. Levinson, M. Lorentzon, B. McKnight, A.P. Morris, M. Muller, J. Suh Ngwa, S. Purcell, S. Rafelt, R.M. Salem, E. Salvi, S. Sanna, J. Shi, U. Sovio, J.R. Thompson, M.C. Turchin, L. Vandenput, D.J. Verlaan, V. Vitart, C.C. White, A. Ziegler, P. Almgren, A.J. Balmforth, H. Campbell, L. Citterio, A. De Grandi, A. Dominiczak, J. Duan, P. Elliott, R. Elosua, J.G. Eriksson, N.B. Freimer, E.J.C. Geus, N. Glorioso, S. Haiqing, A.-L. Hartikainen, A.S. Havulinna, A.A. Hicks, J. Hui, W. Igl, T. Illig, A. Jula, E. Kajantie, T.O. Kilpelainen, M. Koiranen, I. Kolcic, S. Koskinen, P. Kovacs, J. Laitinen, J. Liu, M.-L. Lokki, A. Marusic, A. Maschio, T. Meitinger, A. Mulas, G. Pare, A.N. Parker, J.F. Peden, A. Petersmann, I. Pichler, K.H. Pietilainen, A. Pouta, M. Ridderstrale, J.I. Rotter, J.G. Sambrook, A.R. Sanders, C. Oliver Schmidt, J. Sinisalo, J.H. Smit, H.M. Stringham, G. Bragi Walters, E. Widen, S.H. Wild, G. Willemsen, L. Zagato, L. Zgaga, P. Zitting, H. Alavere, M. Farrall, W.L. McArdle, M. Nelis, M.J. Peters, S. Ripatti, J.B.J. van Meurs, K.K. Aben, K.G. Ardlie, J.S. Beckmann, J.P. Beilby, R.N. Bergman, S. Bergmann, F.S. Collins, D. Cusi, M. den Heijer, G. Eiriksdottir, P.V. Gejman, A.S. Hall, A. Hamsten, H.V. Huikuri, C. Iribarren, M. Kahonen, J. Kaprio, S. Kathiresan, L. Kiemeney, T. Kocher, L.J. Launer, T. Lehtimaki, O. Melander, T.H. Mosley Jr, A.W. Musk, M.S. Nieminen, C.J. O/'Donnell, C. Ohlsson, B. Oostra, L.J. Palmer, O. Raitakari, P.M. Ridker, J.D. Rioux, A. Rissanen, C. Rivolta, H. Schunkert, A.R. Shuldiner, D.S. Siscovick, M. Stumvoll, A. Tonjes, J. Tuomilehto, G.-J. van Ommen, J. Viikari, A.C. Heath, N.G. Martin, G.W. Montgomery, M.A. Province, M. Kayser, A.M. Arnold, L.D. Atwood, E. Boerwinkle, S.J. Chanock, P. Deloukas, C. Gieger, H. Gronberg, P. Hall, A.T. Hattersley, C. Hengstenberg, W. Hoffman, G. Mark Lathrop, V. Salomaa, S. Schreiber, M. Uda, D. Waterworth, A.F. Wright, T.L. Assimes, I. Barroso, A. Hofman, K.L. Mohlke, D.I. Boomsma, M.J. Caulfield, L. Adrienne Cupples, J. Erdmann, C.S. Fox, V. Gudnason, U. Gyllensten, T.B. Harris, R.B. Hayes, M.-R. Jarvelin, V. Mooser, P.B. Munroe, W.H. Ouwehand, B.W. Penninx, P.P. Pramstaller, T. Quertermous, I. Rudan, N.J. Samani, T.D. Spector, H. Volzke, H. Watkins, J.F. Wilson, L.C. Groop, T. Haritunians, F.B. Hu, R.C. Kaplan, A. Metspalu, K.E. North, D. Schlessinger, N.J. Wareham, D.J. Hunter, J.R. O/'Connell, D.P. Strachan, H.E. Wichmann, I.B. Borecki, C.M. van Duijn, E.E. Schadt, U. Thorsteinsdottir, L. Peltonen, A.G. Uitterlinden, P.M. Visscher, N. Chatterjee, R.J.F. Loos, M. Boehnke, M.I. McCarthy, E. Ingelsson, C.M. Lindgren, G.R. Abecasis, K. Stefansson, T.M. Frayling and J.N. Hirschhorn, *Hundreds of variants clustered in genomic loci and biological pathways affect human height.* Nature, 2010. **467**(7317): p. 832-838.

41. Speliotes, E.K., C.J. Willer, S.I. Berndt, K.L. Monda, G. Thorleifsson, A.U. Jackson, H.L. Allen, C.M. Lindgren, J.a. Luan, R. Magi, J.C. Randall, S. Vedantam, T.W. Winkler, L. Qi, T. Workalemahu, I.M. Heid, V. Steinthorsdottir, H.M. Stringham, M.N. Weedon, E. Wheeler, A.R. Wood, T. Ferreira, R.J. Weyant, A.V. Segre, K. Estrada, L. Liang, J. Nemesh, J.-H. Park, S. Gustafsson, T.O. Kilpelainen, J. Yang, N. Bouatia-Naji, T. Esko, M.F. Feitosa, Z. Kutalik, M. Mangino, S. Raychaudhuri, A. Scherag, A.V. Smith, R. Welch, J.H. Zhao, K.K. Aben, D.M. Absher, N. Amin, A.L. Dixon, E. Fisher, N.L. Glazer, M.E. Goddard, N.L. Heard-Costa, V. Hoesel, J.-J. Hottenga, A. Johansson, T. Johnson, S. Ketkar, C. Lamina, S. Li, M.F. Moffatt, R.H. Myers, N. Narisu, J.R.B. Perry, M.J. Peters, M. Preuss, S. Ripatti, F. Rivadeneira, C. Sandholt, L.J. Scott, N.J. Timpson, J.P. Tyrer, S. van Wingerden, R.M. Watanabe, C.C. White, F. Wiklund, C. Barlassina, D.I. Chasman, M.N. Cooper, J.-O. Jansson, R.W. Lawrence, N. Pellikka, I. Prokopenko, J. Shi, E. Thiering, H. Alavere, M.T.S. Alibrandi, P. Almgren, A.M. Arnold, T. Aspelund, L.D. Atwood, B. Balkau, A.J. Balmforth, A.J. Bennett, Y. Ben-Shlomo, R.N. Bergman, S. Bergmann, H. Biebermann, A.I.F. Blakemore, T. Boes, L.L. Bonnycastle, S.R. Bornstein, M.J. Brown, T.A. Buchanan, F. Busonero, H. Campbell, F.P. Cappuccio, C. Cavalcanti-Proenca, Y.-D.I. Chen, C.-M. Chen, P.S. Chines, R. Clarke, L. Coin, J. Connell, I.N.M. Day, M.d. Heijer, J. Duan, S. Ebrahim, P. Elliott, R. Elosua, G. Eiriksdottir, M.R. Erdos, J.G. Eriksson, M.F. Facheris, S.B. Felix, P. Fischer-Posovszky, A.R. Folsom, N. Friedrich, N.B. Freimer, M. Fu, S. Gaget, P.V. Gejman, E.J.C. Geus, C. Gieger, A.P. Gjesing, A. Goel, P. Goyette, H. Grallert, J. Graszler, D.M. Greenawalt, C.J. Groves, V. Gudnason, C. Guiducci, A.-L. Hartikainen, N. Hassanali, A.S. Hall, A.S. Havulinna, C. Hayward, A.C. Heath, C. Hengstenberg, A.A. Hicks, A. Hinney, A. Hofman, G. Homuth, J. Hui, W. Igl, C. Iribarren, B. Isomaa, K.B. Jacobs, I. Jarick, E. Jewell, U. John, T. Jorgensen, P. Jousilahti, A. Jula, M. Kaakinen, E. Kajantie, L.M. Kaplan, S. Kathiresan, J. Kettunen, L. Kinnunen, J.W. Knowles, I. Kolcic, I.R. Konig, S. Koskinen, P. Kovacs, J. Kuusisto, P. Kraft, K. Kvaloy, J. Laitinen, O. Lantieri, C. Lanzani, L.J. Launer, C. Lecoeur, T. Lehtimaki, G. Lettre, J. Liu, M.-L. Lokki, M. Lorentzon, R.N. Luben, B. Ludwig, P. Manunta, D. Marek, M. Marre, N.G. Martin, W.L. McArdle, A. McCarthy, B. McKnight, T. Meitinger, O. Melander, D. Meyre, K. Midthjell, G.W. Montgomery, M.A. Morken, A.P. Morris, R. Mulic, J.S. Ngwa, M. Nelis, M.J. Neville, D.R. Nyholt, C.J. O'Donnell, S. O'Rahilly, K.K. Ong, B. Oostra, G. Pare, A.N. Parker, M. Perola, I. Pichler, K.H. Pietilainen, C.G.P. Platou, O. Polasek, A. Pouta, S. Rafelt, O. Raitakari, N.W. Rayner, M. Ridderstrale, W. Rief, A. Ruokonen, N.R. Robertson, P. Rzehak, V. Salomaa, A.R. Sanders, M.S. Sandhu, S. Sanna, J. Saramies, M.J. Savolainen, S. Scherag, S. Schipf, S. Schreiber, H. Schunkert, K. Silander, J. Sinisalo, D.S. Siscovick, J.H. Smit, N. Soranzo, U. Sovio, J. Stephens, I. Surakka, A.J. Swift, M.-L. Tammesoo, J.-C. Tardif, M. Teder-Laving, T.M. Teslovich, J.R. Thompson, B. Thomson, A. Tonjes, T. Tuomi, J.B.J. van Meurs, G.-J. van Ommen, V. Vatin, J. Viikari, S. Visvikis-Siest, V. Vitart, C.I.G. Vogel, B.F. Voight, L.L. Waite, H. Wallaschofski, G.B. Walters, E. Widen, S. Wiegand, S.H. Wild, G. Willemsen, D.R. Witte, J.C. Witteman, J. Xu, Q. Zhang, L. Zgaga, A. Ziegler, P. Zitting, J.P. Beilby, I.S. Farooqi, J. Hebebrand, H.V. Huikuri, A.L. James, M. Kahonen, D.F. Levinson, F. Macciardi, M.S. Nieminen, C. Ohlsson, L.J. Palmer, P.M. Ridker, M. Stumvoll, J.S. Beckmann, H. Boeing, E. Boerwinkle, D.I. Boomsma, M.J. Caulfield, S.J. Chanock, F.S. Collins, L.A. Cupples, G.D. Smith, J. Erdmann, P. Froguel, H. Gronberg, U. Gyllensten, P. Hall, T. Hansen, T.B. Harris, A.T. Hattersley, R.B. Hayes, J. Heinrich, F.B. Hu, K. Hveem, T. Illig, M.-R. Jarvelin, J. Kaprio, F. Karpe, K.-T. Khaw, L.A. Kiemeney, H. Krude, M. Laakso, D.A. Lawlor, A. Metspalu, P.B. Munroe, W.H. Ouwehand, O. Pedersen, B.W. Penninx, A. Peters, P.P. Pramstaller, T. Quertermous, T. Reinehr, A. Rissanen, I. Rudan, N.J. Samani, P.E.H. Schwarz, A.R. Shuldiner, T.D. Spector, J. Tuomilehto, M. Uda, A. Uitterlinden, T.T. Valle, M. Wabitsch, G. Waeber, N.J. Wareham, H. Watkins, J.F. Wilson, A.F. Wright, M.C. Zillikens, N. Chatterjee, S.A. McCarroll, S. Purcell, E.E. Schadt, P.M. Visscher, T.L. Assimes, I.B. Borecki, P. Deloukas, C.S. Fox, L.C. Groop, T. Haritunians, D.J. Hunter, R.C. Kaplan, K.L. Mohlke, J.R. O'Connell, L. Peltonen, D. Schlessinger, D.P. Strachan, C.M. van Duijn, H.E. Wichmann, T.M. Frayling, U. Thorsteinsdottir, G.R. Abecasis, I. Barroso, M. Boehnke, K. Stefansson, K.E. North, M. I McCarthy, J.N. Hirschhorn, E. Ingelsson and R.J.F. Loos, *Association analyses of 249,796 individuals reveal 18 new loci associated with body mass index.* Nat Genet, 2010. **42**(11): p. 937-948.

42. Schousboe, K., G. Willemsen, K.O. Kyvik, J. Mortensen, D.I. Boomsma, B.K. Cornes, C.J. Davis, C. Fagnani, J. Hjelmborg, J. Kaprio, M. de Lange, M. Luciano, N.G. Martin, N. Pedersen, Pietil, K.H. inen, A. Rissanen, S. Saarni, T.I.A. rensen, G.C.M. van Baal, and J.R. Harris, *Sex Differences in Heritability of BMI: A Comparative Study of Results from Twin Studies in Eight Countries.* Twin Research, 2003. **6**(5): p. 409-421.

43. Willemsen, G., E.J.C. de Geus, M. Bartels, C.E.M. van Beijsterveldt, A.I. Brooks, G.F. Estourgie-van Burk, D.A. Fugman, C. Hoekstra, J.J. Hottenga, K. Kluft, P. Meijer, G.W. Montgomery, P. Rizzu, D. Sondervan, A.B. Smit, S. Spijker, H.E.D. Suchiman, J.A. Tischfield, T. Lehner, P.E. Slagboom, and D.I. Boomsma, *The Netherlands Twin Register Biobank:A Resource for Genetic Epidemiological Studies.* Twin Res Hum Genet., 2010. **13**(3): p. 231-245.

44. Hottenga, J.J., D.I. Boomsma, H.M. Kupper, D. Posthuma, H. Snieder, A.H.M. Willemsen, and E.J.C. De Geus, *Heritability and stability of resting blood pressure.* Twin Res Hum Genet., 2005. **8**: p. 499-508.

45. Kupper, N., G. Willemsen, H. Riese, D. Posthuma, D.I. Boomsma, and E.J.C. de Geus, *Heritability of daytime ambulatory blood pressure in an extended twin design.* Hypertension, 2005. **45**: p. 80-85.

46. Cui, J.S., J.L. Hopper, and S.B. Harrap, *Antihypertensive Treatments Obscure Familial Contributions to Blood Pressure Variation.* Hypertension, 2003. **41**(2): p. 207-210.

47. Palmer, L.J., *Loosening the Cuff: important new advances in modeling antihypertensive treatment effects in genetic studies of hypertension.* Hypertension, 2003. **41**(2): p. 197-198.

48. Friedewald, W.T., R.I. Levy, and D.S. Frerickson, *Estimation of the concentration of low-density lipoprotein cholestrol in plasma, without use of the preparative ultracentrifuge. .* Clinical Chemistry, 1972. **18**: p. 499-502.

49. McCutcheon, A.L., *Latent Class Analysis*. 1987, Beverly Hills: Sage Publications.

50. Ligthart, L., D.R. Nyholt, B.W.J.H. Penninx, and D.I. Boomsma, *The Shared Genetics of Migraine and Anxious Depression.* Headache: The Journal of Head and Face Pain, 2010. **50**(10): p. 1549-1560.
