# Supplementary material for: Sex Differences in Genetic Architecture of Complex Phenotypes?
Source: PLoS One. 2012 Dec 18;7(12):e47371. doi: 10.1371/journal.pone.0047371 (PMC3525575; doi:10.1371/journal.pone.0047371)
Supplement: Table S1 — Lifestyle. (A) Lifestyle Adults. (B) Lifestyle Adolescents (DOC) [file pone.0047371.s002.doc]

**Supplemental Table S1.** Overview of available data for Lifestyle, including source (cohort), mean age, number of subjects, number of complete twin pairs, number of incomplete twins and percentage female participants. The ANTR surveys are part of the longitudinal study to health and personality of the Netherlands Twin Register: For Cohort/survey: 1 = ANTR data collected in 1991, 2= data collected in 1993, 3= data collected in 1995, 4- data collected in 1997, 5= data collected in 2000, 6= data collected in 2002, 7= data collected in 2004, 8= data collected in 2009, bb= bio bank project. For YNTR data the birth cohorts are given. Age (Range) = mean age of the sample and age range. N ss = Number of subjects, N cp = number of complete twin pairs, N icp = number of incomplete twins; Prev = prevalence, ß age = regression coefficient of age on mean/prevalence.

**Table S1A. Lifestyle - Adults**

| **Phenotype** | **Cohort/survey** | **Age (range)** | **N Ss** | **N cp** | **N icp** | % ♀ | **Prev** ♂ | **Prev** ♀ | **ß age** ♂ | **ß age** ♀ |
| --- | --- | --- | --- | --- | --- | --- | --- | --- | --- | --- |
| Ever smoked | 1,2,3,4,5,6,7,8 | 29.3 (18-65) | 10004 | 4290 | 1424 | 64% | 44% | 40% | -.26 | -.28 |
| Current smoking | 1,2,3,4,5,6,7,8 | 29.3 (18-65) | 10004 | 4290 | 1424 | 64% | 28% | 22% | .05 | .005 |
| Nicotine depend | 5,6,7,8 | 1971 (1940-1993)* | 3360 | 852 | 1656 | 66% | 38% | 33% | .05 | -.17 |
| Ever cannabis | 2, 3, 5, 8 | 28.7 (18 – 65) | 7620 | 2641 | 2338 | 65% | 34% | 23% | .16 | 0.17 |
| Regular drinking | 7,8 | 32.0 (18-65) | 7189 | 2540 | 2109 | 68% | 11% | 6% | .09 | -0.005 |
| Alcohol problems 0  1  2  >2 | 2,3,4,5,6,7,8 | 1975 (1930-1992)* | 11697 | 4703 | 2291 | 64% | 61%  21%  11%  7% | 78%  13%  6%  3% | .04  .07  .03 | 0.06  0.11  0.10 |
| Coffee -0-2 cups  -3-5 cups  ->6 cups | 5 | 30.1 (18-65) | 4287 | 1524 | 1239 | 67% | 37%  48%  15% | 63%  21%  16% | -.39  -.31 | -.41  -.32 |
| Exercise | 1,2,3,4,5,6,7,8 | 28.6 (18-65) | 8605 | 3421 | 1763 | 65% | 52% | 49% | .102 | 0.052 |

*birth cohort instead of age because based on longitudinal data

**Table 1B. Lifestyle - A**dolescents

| **Phenotype** | **Cohort/survey** | **Age (range)** | **N Ss** | **N cp** | **N icp** | % ♀ | **Prev** ♂ | **Prev** ♀ | **ß age** ♂ | **ß age** ♀ |
| --- | --- | --- | --- | --- | --- | --- | --- | --- | --- | --- |
| Ever smoked | 1984-1994 | 15.7 (14-19) | 6075 | 2817 | 441 | 56% | 14% | 15% | -.29 | -.27 |
| Current smoking | 1984-1994 | 15.7 (14-19) | 6075 | 2817 | 441 | 56% | 7% | 8% | -.36 | -.29 |
| Ever cannabis | 1984-1994 | 16.2 (13-20) | 6208 | 2826 | 556 | 56% | 14% | 13% | -.66 | -.66 |
| Ever alcohol | 1984-1994 | 15.7 (14-19) | 5917 | 2701 | 515 | 56% | 78% | 75% | -.67 | -.63 |
| Early alcohol initiat | 1984-1994 | 14.2 (13-15) | 3630 | 1649 | 332 | 56% | 75% | 72% | -.08 | -.14 |
| Weekly alcohohol | 1984-1994 | 15.7 (13-18) | 5917 | 2701 | 515 | 56% | 24% | 17% | -.77 | -.55 |
