# Supplementary material for: Sex Differences in Genetic Architecture of Complex Phenotypes?
Source: PLoS One. 2012 Dec 18;7(12):e47371. doi: 10.1371/journal.pone.0047371 (PMC3525575; doi:10.1371/journal.pone.0047371)
Supplement: Table S2 — Emotional and Behavioral problems. (A) Emotional and Behavioral problems - Psychiatry adults (B) Emotional and Behavioral problems - Personality adults. (C) Emotional and Behavioral problems – Internalizing children (D) Emotional and Behavioral problems – Externalizing children (E) Emotional and Behavioral problems – others children (DOC) [file pone.0047371.s003.doc]

**Supplemental S2.** Overview of available data for Emotional and Behavioral Problems, including source (cohort), mean age, number of subjects, number of complete twin pairs, number of incomplete twins and percentage female participants. The ANTR surveys are part of the longitudinal study to health and personality of the Netherlands Twin Register: For Cohort/survey: 1 = ANTR data collected in 1991, 2= data collected in 1993, 3= data collected in 1995, 4- data collected in 1997, 5= data collected in 2000, 6= data collected in 2002, 7= data collected in 2004, 8= data collected in 2009, bb= bio bank project. For YNTR data the birth cohorts are given. Age (Range) = mean age of the sample and age range. N ss = Number of subjects, N cp = number of complete twin pairs, N icp = number of incomplete twins; Prev = prevalence, ß age = regression coefficient of age on mean/prevalence.

**Table 2A. Emotional and Behavioral problems – Psychiatry adults**

| **Phenotype** | **Cohort/survey** | **Age (range)** | **N Ss** | **N cp** | **N icp** | **% ♀** | **Mean (var)**  **♂** | | **Mean (var)** ♀ | | **ß age** ♂ | **ß age** ♀ |
| --- | --- | --- | --- | --- | --- | --- | --- | --- | --- | --- | --- | --- |
| Anxious Dep ASR | 1,3,4,5,6 | 29.5 (18-65) | 7409 | 3238 | 933 | 63% | 17.13 | 9.97 | 22.30 | 9.60 | -.16 | -.54 |
| Anxiety | 1,2,4,5,6 | 29.6 (18-65) | 7463 | 3261 | 941 | 63% | 32.08 | 9.17 | 35.23 | 7.99 | -.21 | -.52 |
| OCS | 6,8 | 31.7 (18-65) | 7128 | 2499 | 2130 | 68% | 7.38 | 5.41 | 7.20 | 5.36 | -.56 | -.66 |
| Borderline | 7,8 | 31.9 (18-65) | 7085 | 2508 | 2069 | 69% | 3.71 | 1.02 | 3.90 | 1.06 | -.15 | -.19 |
| Anger | 1,2,7 | 30.71 (18-65) | 5744 | 2438 | 868 | 64% | 15.60 | 4.49 | 15.74 | 4.23 | -.58 | -.68 |
| ADHD | 7,8 | 31.2 (18-65) | 6327 | 2045 | 2237 | 68% | 8.03 | 4.19 | 8.14 | 3.85 | -.41 | -.41 |
| **Phenotype** | **Cohort/survey** | **Age (range)** | **N Ss** | **N cp** | **N icp** | % ♀ | **Prev** ♂ | | **Prev** ♀ | | **ß age** ♂ | **ß age** ♀ |
| Social Fear | 4, 5 | 29.0 (18-65) | 5170 | 2053 | 1064 | 66% | 62% | | 44% | | .09 | .06 |
| Blood Fear | 4,5 | 29.0 (18-65) | 5170 | 2053 | 1064 | 66% | 50% | | 53% | | .09 | .12 |
| Loneliness | 7,8 | 31.0 (18-65) | 8380 | 3079 | 2222 | 68% | 52%  22%  8%  18% | | 45%  29%  12%  14% | | .067  .047  .007 | .094  .045  .045 |

Cohort: 1 = 1991, 2=1993, 3=1995, 4-1997, 5=2000, 6=2002, 7=2004, 8=2009

**Table 2B. Emotional and Behavioral problems - Personality adults**

| **Phenotype** | **Cohort/survey** | **Age (range)** | **N Ss** | **N cp** | **N icp** | **% ♀** | **Mean (var)**  **♂** | | **Mean (var)** ♀ | | **ß age** ♂ | **ß age** ♀ |
| --- | --- | --- | --- | --- | --- | --- | --- | --- | --- | --- | --- | --- |
| Extraversion | 7 | 34.1 (18-65) | 4731 | 1624 | 1483 | 70% | 27.64 | 7.07 | 30.96 | 7.56 | -.19 | -.46 |
| Neuroticism | 7 | 34.1 (18-65) | 4731 | 1624 | 1483 | 70% | 42.67 | 5.61 | 42.29 | 5.88 | -1.00 | -.65 |
| Conscientiousness | 7 | 34.1 (18-65) | 4731 | 1624 | 1483 | 70% | 45.04 | 5.15 | 45.44 | 5.51 | .28 | -.02 |
| Agreeableness | 7 | 34.1 (18-65) | 4731 | 1624 | 1483 | 70% | 43.26 | 4.60 | 45.88 | 4.81 | .74 | .29 |
| Openness to Experience | 7 | 34.1 (18-65) | 4731 | 1624 | 1483 | 70% | 36.68 | 5.65 | 37.07 | 5.91 | -.19 | -.18 |
| Thrill & adventure Seeking | 1,2,4,5,6 | 28.5 (18-65) | 7222 | 3108 | 1006 | 63% | 39.20 | 9.63 | 32.59 | 9.63 | -3.39 | -4.37 |
| Disinhibition | 1,2,4,5,6 | 28.5 (18-65) | 7222 | 3108 | 1006 | 63% | 34.87 | 7.76 | 28.91 | 7.29 | -1.86 | -2.03 |
| Experience seeking | 1,2,4,5,6 | 28.5 (18-65) | 7222 | 3108 | 1006 | 63% | 35.10 | 8.22 | 32.64 | 8.09 | -0.43 | -0.87 |
| Boredom susceptibility | 1,2,4,5,6 | 28.5 (18-65) | 7222 | 3108 | 1006 | 63% | 37.17 | 7.21 | 35.01 | 7.47 | -.79 | -.83 |
| Sensation seeking | 1,2,4,5,6 | 28.5 (18-65) | 7222 | 3108 | 1006 | 63% | 11.54 | 1.88 | 10.15 | 1.82 | -.53 | -.66 |

Cohort: 1 = 1991, 2=1993, 3=1995, 4-1997, 5=2000, 6=2002, 7=2004, 8=2009

**Table 2C. Emotional and Behavioral problems – Internalizing children**

| **Phenotype** | **Cohort/survey** | **Age (range)** | **N Ss** | **N cp** | **N icp** | **% ♀** | **Mean (var)**  **♂** | | **Mean (var)** ♀ | | **ß age** ♂ | **ß age** ♀ |
| --- | --- | --- | --- | --- | --- | --- | --- | --- | --- | --- | --- | --- |
| Anxious Depr – 3 | 1986-2004 | 3.3 (2.4-5.0) | 31864 | 15873 | 118 | 50% | 3.49 | 2.85 | 3.33 | 2.85 | NA | NA |
| Anxious Depr -7 | 1986 - 99 | 7.4 (6.1-9.8) | 10181 | 10106 | 75 | 50% | 2.02 | 2.54 | 2.14 | 2.47 | NA | NA |
| Anxious Depr -10 | 1986-1997 | 10.1 (8.7-12.9) | 6970 | 6927 | 43 | 48% | 2.30 | 2.94 | 2.41 | 2.79 | NA | NA |
| Anxious Depr -12 | 1986-1998 | 12.2 (11.2-14.3) | 6554 | 6516 | 38 | 49% | 2.02 | 2.81 | 2.18 | 2.81 | NA | NA |
| Internalizing -3 | 1986-2004 | 3.3 (2.4-5.0) | 31864 | 15873 | 118 | 50% | 4.81 | 3.66 | 4.43 | 3.84 | NA | NA |
| Internalizing – 7 | 1986 - 99 | 7.4 (6.1-9.8) | 10181 | 10106 | 75 | 49% | 4.18 | 4.23 | 4.44 | 4.15 | NA | NA |
| Internalizing -10 | 1986-1997 | 10.1 (8.7-12.9) | 6970 | 6927 | 43 | 48% | 4.45 | 4.74 | 4.69 | 4.50 | NA | NA |
| Internalizing -12 | 1986-1998 | 12.2 (11.2-14.3) | 6554 | 6516 | 38 | 49% | 3.94 | 4.61 | 4.17 | 4.61 | NA | NA |
| Somatic compl- 3 | 1986-2004 | 3.3 (2.4-5.0) | 31864 | 15873 | 118 | 50% | 0.63 | 3.74 | 0.70 | 3.66 | NA | NA |
| Somatic compl - 7 | 1986 - 99 | 7.4 (6.1-9.8) | 10181 | 10106 | 75 | 49% | 0.76 | 1.29 | 0.89 | 1.17 | NA | NA |
| Somatic compl - 10 | 1986-1997 | 10.1 (8.7-12.9) | 6970 | 6927 | 43 | 48% | 0.76 | 0.14 | 0.94 | 1.21 | NA | NA |
| Somatic compl - 12 | 1986-1998 | 12.2 (11.2-14.3) | 6554 | 6516 | 38 | 49% | 0.66 | 1.26 | 0.81 | 1.17 | NA | NA |
| Withdrawn - 3 | 1986-2004 | 3.3 (2.4-5.0) | 31864 | 15873 | 118 | 50% | 1.23 | 1.31 | 1.08 | 1.48 | NA | NA |
| Withdrawn - 7 | 1986 - 99 | 7.4 (6.1-9.8) | 10181 | 10106 | 75 | 49% | 1.50 | 1.52 | 1.51 | 1.60 | NA | NA |
| Withdrawn - 10 | 1986-1997 | 10.1 (8.7-12.9) | 6970 | 6927 | 43 | 48% | 1.51 | 1.65 | 1.46 | 1.71 | NA | NA |
| Withdrawn - 12 | 1986-1998 | 12.2 (11.2-14.3) | 6554 | 6516 | 38 | 49% | 1.35 | 1.62 | 1.27 | 1.75 | NA | NA |

**Table 2D Emotional and Behavioral problems – Externalizing children**

| **Phenotype** | **Cohort/survey** | **Age (range)** | **N Ss** | **N cp** | **N icp** | **% ♀** | **Mean (var)**  **♂** | | **Mean (var)** ♀ | | **ß age** ♂ | **ß age** ♀ |
| --- | --- | --- | --- | --- | --- | --- | --- | --- | --- | --- | --- | --- |
| Externalizing -3 | 1986-2004 | 3.3 (2.4-5.0) | 31864 | 15873 | 118 | 50% | 16.4 | 8.88 | 14.6 | 9.69 | NA | NA |
| Externalizing -7 | 1986 - 99 | 7.4 (6.1-9.8) | 10181 | 10106 | 75 | 49% | 8.36 | 6.12 | 5.59 | 6.75 | NA | NA |
| Externalizing -10 | 1986-1997 | 10.1 (8.7-12.9) | 6970 | 6927 | 43 | 48% | 7.60 | 5.38 | 5.40 | 6.86 | NA | NA |
| Externalizing -12 | 1986-1998 | 12.2 (11.2-14.3) | 6554 | 6516 | 38 | 49% | 6.22 | 4.50 | 4.88 | 6.26 | NA | NA |
| Aggression -3 | 1986-2004 | 3.3 (2.4-5.0) | 31864 | 15873 | 118 | 50% | 3.70 | 2.50 | 2.17 | 2.75 | NA | NA |
| Aggression -7 | 1986 - 99 | 7.4 (6.1-9.8) | 10181 | 10106 | 75 | 49% | 7.12 | 5.22 | 4.73 | 5.63 | NA | NA |
| Aggression -10 | 1986-1997 | 10.1 (8.7-12.9) | 6970 | 6927 | 43 | 48% | 6.42 | 4.59 | 4.51 | 5.62 | NA | NA |
| Aggression - 12 | 1986-1998 | 12.2 (11.2-14.3) | 6554 | 6516 | 38 | 49% | 5.24 | 3.85 | 4.08 | 5.11 | NA | NA |
| Opposition def - 3 | 1986-2004 | 3.3 (2.4-5.0) | 31864 | 15873 | 118 | 50% | 9.90 | 9.75 | 5.99 | 6.14 | NA | NA |
| Rule-breaking beh - 7 | 1986 - 99 | 7.4 (6.1-9.8) | 10181 | 10106 | 75 | 49% | 1.34 | 1.00 | 1.87 | 1.53 | NA | NA |
| Rule-breaking beh – 10 | 1986-1997 | 10.1 (8.7-12.9) | 6970 | 6927 | 43 | 48% | 1.21 | 0.82 | 1.23 | 1.62 | NA | NA |
| Rule-breaking beh- 12 | 1986-1998 | 12.2 (11.2-14.3) | 6554 | 6516 | 38 | 49% | 1.03 | 0.67 | 1.15 | 1.59 | NA | NA |

**Table 2E. Emotional and Behavioral problems – other scales children**

| **Phenotype** | **Cohort/survey** | **Age (range)** | **N Ss** | **N cp** | **N icp** | **% ♀** | **Mean (var)**  **♂** | | **Mean (var)**  ♀ | | **ß age** ♂ | **ß age** ♀ |
| --- | --- | --- | --- | --- | --- | --- | --- | --- | --- | --- | --- | --- |
| Social problems - 7 | 1986 - 99 | 7.4 (6.1-9.8) | 10181 | 10106 | 75 | 49% | 1.32 | 1.48 | 1.10 | 1.61 | NA | NA |
| Social problems - 10 | 1986-1997 | 10.1 (8.7-12.9) | 6970 | 6927 | 43 | 48% | 1.43 | 1.66 | 1.00 | 1.53 | NA | NA |
| Social problems - 12 | 1986-1998 | 12.2 (11.2-14.3) | 6554 | 6516 | 38 | 49% | 1.35 | 1.57 | 0.82 | 1.62 | NA | NA |
| Thought problems - 7 | 1986 - 99 | 7.4 (6.1-9.8) | 10181 | 10106 | 75 | 49% | 0.47 | 1.05 | 0.67 | 1.59 | NA | NA |
| Thought problems - 10 | 1986-1997 | 10.1 (8.7-12.9) | 6970 | 6927 | 43 | 48% | 0.43 | 0.82 | 1.10 | 1.61 | NA | NA |
| Thought problems - 12 | 1986-1998 | 12.2 (11.2-14.3) | 6554 | 6516 | 38 | 49% | 0.38 | 0.73 | 1.22 | 1.81 | NA | NA |
| Sleep problems - 3 | 1986-2004 | 3.3 (2.4 | 31864 | 15873 | 118 | 50% | 2.04 | 2.64 | 1.06 | 1.82 | NA | NA |
| Total problems - 3 | 1986-2004 | 3.3 (2.4 | 31864 | 15873 | 118 | 50% | 32.41 | 16.38 | 19.08 | 17.74 | NA | NA |
| Overactive -3 | 1986-2004 | 3.3 (2.4-5.0) | 31864 | 15873 | 118 | 50% | 2.88 | 2.00 | 0.33 | 0.95 | NA | NA |
| Attention problems -7 | 1986 - 99 | 7.4 (6.1-9.8) | 10181 | 10106 | 75 | 49% | 3.24 | 2.42 | 0.27 | 0.95 | NA | NA |
| Attention problems- 10 | 1986-1997 | 10.1 (8.7-12.9) | 6970 | 6927 | 43 | 48% | 3.29 | 2.45 | 2.03 | 2.64 | NA | NA |
| Attention problems-12 | 1986-1998 | 12.2 (11.2-14.3) | 6554 | 6516 | 38 | 49% | 2.97 | 2.25 | 1.98 | 2.87 | NA | NA |
