# Supplementary material for: Sex Differences in Genetic Architecture of Complex Phenotypes?
Source: PLoS One. 2012 Dec 18;7(12):e47371. doi: 10.1371/journal.pone.0047371 (PMC3525575; doi:10.1371/journal.pone.0047371)
Supplement: Table S3 — Brain and Cognition (DOC) [file pone.0047371.s004.doc]

**Table S3. Brain and Cognition.** Overview of available data, including source (cohort), mean age, number of subjects, number of complete twin pairs, number of incomplete twins and percentage female participants. The ANTR surveys are part of the longitudinal study to health and personality of the Netherlands Twin Register: For Cohort/survey: 1 = ANTR data collected in 1991, 2= data collected in 1993, 3= data collected in 1995, 4- data collected in 1997, 5= data collected in 2000, 6= data collected in 2002, 7= data collected in 2004, 8= data collected in 2009, bb= bio bank project. For YNTR data the birth cohorts are given. Age (Range) = mean age of the sample and age range. N ss = Number of subjects, N cp = number of complete twin pairs, N icp = number of incomplete twins; Prev = prevalence, ß age = regression coefficient of age on mean/prevalence.

| **Phenotype** | **Cohort/survey** | **Age (range)** | **N Sub-jects** | **N comp pairs** | **N incomp** | % ♀ | **Mean (var)**  **♂** | | **Mean (var)**  ♀ | | **ß age** ♂ | **ß age** ♀ |
| --- | --- | --- | --- | --- | --- | --- | --- | --- | --- | --- | --- | --- |
| F3 power | 1986-1988 | 5.3 (4.9 – 5.9) | 366 | 165 | 51 | 51% | 11.0 | 2.51 | 11.7 | 2.27 | .25 | -.04 |
| F3 power | 1986-1988 | 6.8 (6.4 – 7.5) | 378 | 186 | 49 | 49% | 11.5 | 2.46 | 12.1 | 2.49 | .12 | -.13 |
| F3 power | 1974-1977 | 16.1 (14.8-18.0) | 426 | 213 | 54 | 54% | 8.2 | 3.58 | 8.5 | 3.36 | -.35 | -.61 |
| F3 power | 1974-1977 | 17.6 (16.4-19.5) | 387 | 191 | 53 | 53% | 7.5 | 3.49 | 8.8 | 3.39 | -.32 | .60 |
| F3 power | 1955-1986 | 25.7 (18.7-33.9) | 393 | 131 | 54 | 54% | 5.3 | 3.60 | 6.8 | 4.15 | -.33 | -.56 |
| F3 power | 1929-1955 | 49.3 (36.0 – 71.0) | 349 | 119 | 61 | 61% | 3.7 | 3.68 | 5.6 | 4.05 | -1.29 | -.05 |
| Educational level 12 | 1986-1996 | 11.92 (10.5-13.5) | 8790 | 4074 | 642 | 53% | 538.0 | 8.62 | 537.0 | 8.78 | NA | NA |
| Educational attainment | 1,2,3,4,5,6,7,8 | 32.23 (21.0-65.0) | 7342 | 2874 | 1594 | 65% | 4.90 | 1.57 | 4.90 | 1.50 | .27 | -.09 |
| Educational attainment | 1,2,3,4,5,6,7,8 | 31.92 (21.0-65.0) | 6952 | 2571 | 1810 | 65% | 109.4 | 14.5 | 109.5 | 14.1 | NA | NA |
| IQ age 5 | 1986-88; 90-91 | 5.5 (4.9 -5.9) | 889 | 444 | 1 | 50% | 103.2 | 14.6 | 102.6 | 14.7 | NA | NA |
| IQ age 7 | 1986-88 | 6.8 (6.4-7.5) | 382 | 191 | 0 | 50% | 108.3 | 14.6 | 105.6 | 16.1 | NA | NA |
| IQ age 10 | 1986-88 | 10.0 (9.1-10.9) | 392 | 196 | 0 | 50% | 98.3 | 14.1 | 101.3 | 13.7 | NA | NA |
| IQ age 12 | 1986-88; 90-91 | 12.2 (11.9-13.1) | 734 | 366 | 2 | 52% | 110.0 | 14.6 | 108.6 | 14.8 | NA | NA |
| IQ age 18 | 1986-88; 76-77 | 17.9 (16.4-19.5) | 758 | 375 | 8 | 53% | 11.0 | 2.51 | 11.7 | 2.27 | .25 | -.04 |

Cohort: 1 = 1991, 2=1993, 3=1995, 4-1997, 5=2000, 6=2002, 7=2004, 8=2009, Age xx represents age-cohorts from the Y-NTR
