# Supplementary material for: Sex Differences in Genetic Architecture of Complex Phenotypes?
Source: PLoS One. 2012 Dec 18;7(12):e47371. doi: 10.1371/journal.pone.0047371 (PMC3525575; doi:10.1371/journal.pone.0047371)
Supplement: Table S4 — BMI and Height. (A) BMI and Height – adult (B) BMI and Height – children and adolescents (DOC) [file pone.0047371.s005.doc]

**Supplemental Table S4.** Overview of available data for BMI and Height, including source (cohort), mean age, number of subjects, number of complete twin pairs, number of incomplete twins and percentage female participants. The ANTR surveys are part of the longitudinal study to health and personality of the Netherlands Twin Register: For Cohort/survey: 1 = ANTR data collected in 1991, 2= data collected in 1993, 3= data collected in 1995, 4- data collected in 1997, 5= data collected in 2000, 6= data collected in 2002, 7= data collected in 2004, 8= data collected in 2009, bb= bio bank project. For YNTR data the birth cohorts are given. Age (Range) = mean age of the sample and age range. N ss = Number of subjects, N cp = number of complete twin pairs, N icp = number of incomplete twins; Prev = prevalence, ß age = regression coefficient of age on mean/prevalence.

**Table 4A. BMI and Height - adults**

| **Phenotype** | **Cohort/survey** | **Age (range)** | **N Ss** | **N cp** | **N icp** | % ♀ | **Mean (var)**  **♂** | | **Mean (var)**  ♀ | | **ß age** ♂ | **ß age** ♀ |
| --- | --- | --- | --- | --- | --- | --- | --- | --- | --- | --- | --- | --- |
| BMI adults | 1,2,3,4,5,6,7,8 | 28.77 (18-65) | 11370 | 4725 | 1920 | 64% | 22.95 | 2.86 | 22.67 | 3.48 | 1.36 | 1.07 |
| Height | 1,2,3,4,5,6,7,8, bb | 28.77 (18-65) | 11340 | 4710 | 1920 | 64% | 182.8 | 7.16 | 169.5 | 6.43 | .87 | .83 |

Cohort: 1 = 1991, 2=1993, 3=1995, 4-1997, 5=2000, 6=2002, 7=2004, 8=2009, bb=biobank

**Table 4B. Birthweight, BMI and height - children and adolescents**

| **Phenotype** | **Cohort/survey** | **Age (range)** | **N Ss** | **N cp** | **N icp** | % ♀ | **Mean (var)**  **♂** | | **Mean (var)**  ♀ | | **ß age** ♂ | **ß age** ♀ |
| --- | --- | --- | --- | --- | --- | --- | --- | --- | --- | --- | --- | --- |
| Birth weight | 1986-2004 | 36.6 (24-42)* | 34617 | 17277 | 63 | 50% | 2557 | 385 | 2451 | 369 | 399 | 385 |
| BMI age 3 | 1986-2002 | 3.0 (2.5-3.5) | 20828 | 10360 | 108 | 50% | 15.74 | 1.23 | 15.48 | 1.31 | -.11 | -.06 |
| BMI age 5 | 1986-2000 | 5.1 (4-6) | 9841 | 4896 | 78 | 50% | 15.05 | 1.24 | 14.95 | 1.53 | -.04 | -.07 |
| BMI age 7 | 1986-1998 | 7.4 (6-8) | 12154 | 6067 | 49 | 51% | 15.28 | 1.65 | 15.38 | 1.92 | .05 | .15 |
| BMI age 10 | 1986-1995 | 10.1 (9-11) | 8764 | 4366 | 20 | 51% | 16.30 | 2.07 | 16.52 | 2.28 | .00 | .13 |
| BMI age 12 | 1985-1993 | 12.1 (11-13) | 6234 | 3099 | 36 | 51% | 17.21 | 2.35 | 17.51 | 2.53 | .23 | .17 |
| BMI age 14 | 1990-1994 | 14.7 (13.9-16) | 3275 | 1549 | 177 | 55% | 18.96 | 2.30 | 19.41 | 2.62 | .37 | .35 |
| BMI age 16 | 1988-1992 | 16.8 (15-18) | 2256 | 1085 | 105 | 57% | 20.30 | 2.37 | 20.66 | 2.77 | .20 | .13 |
| Height age 1 | 1986-2003 | 1 (.6-1.3) | 31829 | 15761 | 307 | 50% | 61.63 | 2.77 | 59.75 | 2.74 | 13.69 | 14.08 |
| Height age 2 | 1986-2003 | 2 (1.6-2.3) | 23147 | 11470 | 207 | 50% | 87.62 | 3.40 | 86.50 | 3.34 | 1.58 | 1.64 |
| Height age 3 | 1986-2002 | 3 (2.5-3.5) | 21120 | 10521 | 78 | 50% | 97.28 | 3.82 | 96.34 | 3.83 | 1.50 | 1.52 |
| Height age 5 | 1986-2000 | 5.1 (4-6) | 10187 | 5079 | 29 | 50% | 111.04 | 4.87 | 110.33 | 4.87 | 6.36 | 6.40 |
| Height age 7 | 1986-1998 | 7.4 (6-8) | 12331 | 6160 | 11 | 51% | 128.56 | 5.70 | 127.85 | 5.66 | 2.25 | 2.42 |
| Height age 10 | 1986-1995 | 10.1 (9-11) | 8917 | 4448 | 19 | 51% | 143.97 | 6.54 | 143.60 | 6.75 | 1.89 | 2.16 |
| Height age 12 | 1985-1993 | 12.1 (11-13) | 6344 | 3161 | 21 | 51% | 154.91 | 7.38 | 155.95 | 7.35 | 1.94 | 2.00 |
| Height age 14 | 1990-1994 | 14.7 (13.9-16) | 3374 | 1630 | 114 | 55% | 172.79 | 8.32 | 167.13 | 6.41 | 2.5 | .74 |
| Height age 16 | 1988-1992 | 16.8 (15-18) | 2358 | 1137 | 83 | 57% | 181.24 | 7.08 | 169.59 | 6.40 | .47 | .27 |
