# Supplementary material for: Sex Differences in Genetic Architecture of Complex Phenotypes?
Source: PLoS One. 2012 Dec 18;7(12):e47371. doi: 10.1371/journal.pone.0047371 (PMC3525575; doi:10.1371/journal.pone.0047371)
Supplement: Table S6 — Number of twin pairs required to detect significant difference between correlation in DZ same sex (DZss) twin pairs and DZ opposite sex (DZos) twin pairs. (DOC) [file pone.0047371.s007.doc]

**Supplemental Table S6.** Number of twin pairs required to detect significant difference between correlation in DZ same sex (DZss) twin pairs and DZ opposite sex (DZos) twin pairs.

|  |  | Power of the test at the significance level of 0.05 (with 1df) | | | | |
| --- | --- | --- | --- | --- | --- | --- |
| RDzss/Rdzos |  | **.75** | **.80** | **.90** | **.95** | **.99** |
|  | .55/.225 | 212 | 240 | 321 | 397 | 561 |
|  | .50/.25 | 284 | 321 | 430 | 532 | 752 |
|  | .45/.225 | 383 | 433 | 580 | 718 | 1012 |
|  | .40/.20 | 525 | 594 | 795 | 984 | 1390 |
|  | .35/.175 | 735 | 831 | 1112 | 1375 | 1944 |
|  | .30/.15 | 1060 | 1198 | 1604 | 1984 | 2805 |
|  | .25/.125 | 1602 | 1812 | 2425 | 2999 | 4241 |
|  | .20/.10 | 2603 | 2944 | 3941 | 4875 | 6892 |
|  | .15/.075 | 4771 | 5396 | 7223 | 8933 | 12630 |
