# Supplementary material for: Sex Differences in Genetic Architecture of Complex Phenotypes?
Source: PLoS One. 2012 Dec 18;7(12):e47371. doi: 10.1371/journal.pone.0047371 (PMC3525575; doi:10.1371/journal.pone.0047371)
Supplement: Table S7 — Significantly different DZ correlations (A) Twin correlations for traits with significantly lower DZ opposite-sex correlations than DZ same-sex correlations. For these traits, a genetic model was fitted to the data to test whether the difference was due to different genes being expressed in men and women or environmental factors being less correlated in opposite-sex pairs (see Supplementary Table 11C and D for results). (B) Full model including additive genetic, common and unique environmental factors (a2, c2, and e2 give explained variance for traits with evidence for qualitative sex differences; γ and φ represent respectively the genetic correlation and environmental correlation in DZ-opposite sex twin pairs (C) Parameter estimates based on most parsimonious model (D) Overview of the twin correlations for traits where DZ correlations were significantly different from each other (but Rdos not lower than Rdzm/Rdzf). No additional models were fitted. (DOC) [file pone.0047371.s008.doc]

**Supplementary Table S7A. Twin correlations for traits with significantly lower DZ opposite-sex correlations than DZ same-sex correlations. For these traits, a genetic model was fitted to the data to test whether the difference was due to different genes being expressed in men and women or environmental factors being less correlated in opposite-sex pairs (see Supplementary Table 11C and D for results).**

|  | **Model 1: 5 correlations (R)** | | | | | **Best model** | | |
| --- | --- | --- | --- | --- | --- | --- | --- | --- |
|  | Rmzm | Rdzm | Rmzf | Rdzf | Rdos | Rdzm | Rdzf | Rdos |
| **Lifestyle -Adolescents** | | | | | | | | |
| Ever alcohol 14-18 year | 0.82 | 0.69 | 0.91 | 0.77 | 0.61 | 0.73 | 0.73 | **0.61** |
| Weekly alcohol use 14-18 year | 0.70 | 0.64 | 0.74 | 0.66 | 0.46 | 0.65 | 0.65 | **0.46** |
| **Personality and Psychopathology – Personality adults** | | | | | | | | |
| Thrill & adventure adults | 0.67 | 0.4 | 0.61 | 0.38 | 0.23 | 0.39 | 0.39 | **0.23** |
| Sensation seeking adults | 0.62 | 0.45 | 0.61 | 0.39 | 0.29 | 0.41 | 0.41 | **0.29** |
| **BMI and Height – children and adolescents** | | | | | | | | |
| Height -7 | 0.94 | 0.6 | 0.93 | 0.58 | 0.54 | 0.59 | 0.59 | **0.54** |
| **Metabolic risk factors & Migraine** | | | | | | | | |
| HDL | 0.70 | 0.51 | 0.62 | 0.36 | 0.21 | 0.40 | 0.40 | **0.21** |

**Supplementary Table S7B. Full model including additive genetic, common and unique environmental factors (a^2^ , c^2^ , and e^2^ give explained variance for traits with evidence for qualitative sex differences; γ and φ represent respectively the genetic correlation and environmental correlation in DZ-opposite sex twin pairs**

|  | a^2^ M | a^2^ F | c^2^ M | c^2^ F | e^2^ M | e^2^ F | R_g_dz-os (γ) | R_c_dz-os (φ) |
| --- | --- | --- | --- | --- | --- | --- | --- | --- |
| **Lifestyle -Adolescents** | | | | | | | | |
| Ever alcohol 14-18 | 0.27 | 0.27 | 0.56 | 0.63 | 0.18 | 0.09 |  | 0.80 |
| Weekly alc 14-18 | 0.12 | 0.14 | 0.58 | 0.59 | 0.30 | 0.27 |  | 0.67 |
| **Personality and Psychopathology – Personality adults** | | | | | | | | |
| Thrill & adventure | 0.54 | 0.47 | 0.13 | 0.15 | 0.33 | 0.39 | 0.18 |  |
| Sensation seeking | 0.35 | 0.45 | 0.27 | 0.16 | 0.38 | 0.39 | 0.19 |  |
| **Height** | | | | | | | | |
| Height -7 years | 0.68 | 0.70 | 0.26 | 0.23 | 0.06 | 0.07 | 0.43 |  |
| **Metabolic risk factors** | | | | | | | | |
| HDL | 0.38 | 0.54 | 0.32 | 0.09 | 0.29 | 0.38 | 0.08 |  |

**Supplementary Table S7C. Parameter estimates based on most parsimonious model**

|  | a^2^ M | a^2^ F | c^2^ M | c^2^ F | e^2^ M | e^2^ F | R_g_dos (γ) | R_c_dos (φ) |
| --- | --- | --- | --- | --- | --- | --- | --- | --- |
| **Lifestyle -Adolescents** | | | | | | | | |
| Ever alcohol 14-18 | 0.28 | | 0.59 | | 0.12 | |  | 0.79 |
| Weekly alc 14-18 | 0 | | 0.69 | | 0.31 | |  | 0.66 |
| **Personality and Psychopathology – Personality adults** | | | | | | | | |
| Thrill & adventure | 0.64 | | 0 | | 0.36 | | 0.36 |  |
| Sensation seeking | 0.41 | | 0.20 | | 0.39 | | 0.20 |  |
| **Height** | | | | | | | | |
| Height -7 years | 0.69 | | 0.25 | | 0.06 | | 0.43 |  |
| **Metabolic risk factors** | | | | | | | | |
| HDL | 0.70 | 0.62 | 0 | 0 | 0.30 | 0.38 | 0.50 |  |

**Supplementary Table S7D.** Overview of the twin correlations for traits where DZ correlations were significantly different from each other (but Rdos not lower than Rdzm/Rdzf). No additional models were fitted.

|  | **Model 1** | | | | | **Best model** | | |
| --- | --- | --- | --- | --- | --- | --- | --- | --- |
|  | Rmzm | Rdzm | Rmzf | Rdzf | Rdos | Rdzm | Rdzf | Rdos |
| **Personality and Psychopathology – Internalizing behavior children** | | | | | | | | |
| Anxious Depr – 3 | 0.73 | 0.33 | 0.72 | 0.32 | 0.38 | 0.33 | 0.32 | **0.38** |
| Anxious Depr -7 | 0.67 | 0.32 | 0.66 | 0.3 | 0.39 | 0.31 | 0.31 | **0.39** |
| Anxious Depr -10 | 0.66 | 0.27 | 0.62 | 0.32 | 0.38 | 0.29 | 0.29 | **0.38** |
| Anxious Depr -12 | 0.64 | 0.24 | 0.64 | 0.33 | 0.42 | **0.24** | **0.32** | **0.42** |
| Internalizing -3 | 0.77 | 0.43 | 0.76 | 0.42 | 0.46 | 0.42 | 0.42 | **0.46** |
| Internalizing – 7 | 0.73 | 0.41 | 0.73 | 0.45 | 0.51 | 0.43 | 0.43 | **0.51** |
| Internalizing -10 | 0.66 | 0.38 | 0.7 | 0.44 | 0.49 | 0.41 | 0.41 | **0.49** |
| Internalizing -12 | 0.73 | 0.38 | 0.7 | 0.45 | 0.52 | 0.42 | 0.42 | **0.52** |
| Somatic complaints, CBCL - 3 | 0.93 | 0.88 | 0.95 | 0.93 | 0.93 | **0.88** | **0.93** | **0.93** |
| Somatic complaints, CBCL - 7 | 0.54 | 0.28 | 0.63 | 0.41 | 0.37 | **0.28** | **0.41** | **0.37** |
| Somatic complaints, CBCL - 10 | 0.53 | 0.23 | 0.63 | 0.34 | 0.33 | **0.23** | **0.34** | **0.33** |
| Somatic complaints, CBCL - 12 | 0.52 | 0.36 | 0.63 | 0.42 | 0.44 | 0.39 | 0.39 | **0.44** |
| Withdrawn, CBCL - 3 | 0.73 | 0.41 | 0.76 | 0.46 | 0.48 | **0.41** | **0.46** | **0.48** |
| Withdrawn, CBCL - 7 | 0.69 | 0.26 | 0.67 | 0.33 | 0.35 | **0.26** | **0.33** | **0.35** |
| Withdrawn, CBCL - 10 | 0.67 | 0.33 | 0.67 | 0.33 | 0.39 | 0.33 | 0.33 | **0.38** |
| Withdrawn, CBCL - 12 | 0.7 | 0.32 | 0.65 | 0.3 | 0.42 | 0.31 | 0.31 | **0.42** |
| **Personality and Psychopathology – Externalizing behavior children** | | | | | | | | |
| Externalizing -12 | 0.85 | 0.46 | 0.81 | 0.48 | 0.55 | 0.47 | 0.47 | **0.55** |
| Aggress beh - 12 | 0.83 | 0.44 | 0.79 | 0.45 | 0.51 | 0.44 | 0.44 | **0.51** |
| Overactive -3 | 0.71 | 0.15 | 0.69 | 0.17 | 0.24 | 0.16 | 0.16 | **0.24** |
| Rule-breaking behavior, CBCL - 12 | 0.79 | 0.49 | 0.83 | 0.53 | 0.63 | 0.51 | 0.51 | **0.62** |
| **Personality and Psychopathology – Other** | | | | | | | | |
| Social problems, CBCL - 7 | 0.76 | 0.36 | 0.74 | 0.29 | 0.34 | **0.36** | **0.29** | **0.34** |
| Social problems, CBCL - 12 | 0.75 | 0.21 | 0.72 | 0.24 | 0.3 | 0.28 | 0.28 | **0.33** |
| Thought problems, CBC - 10 | 0.58 | 0.18 | 0.45 | 0.2 | 0.26 | 0.19 | 0.19 | **0.26** |
| **Brain and cognition** | | | | | | | | |
| Education adults | 0.69 | 0.38 | 0.68 | 0.53 | 0.39 | **0.38** | **0.53** | **0.39** |
| **BMI and height – children and adolescents** | | | | | | | | |
| BMI – 14 | 0.82 | 0.35 | 0.83 | 0.49 | 0.35 | **0.35** | **0.49** | **0.35** |
