# Supplementary material for: Sex Differences in Genetic Architecture of Complex Phenotypes?
Source: PLoS One. 2012 Dec 18;7(12):e47371. doi: 10.1371/journal.pone.0047371 (PMC3525575; doi:10.1371/journal.pone.0047371)
Supplement: Table S8 — Overview of all published GWA studies for Height based on the database published on www.genome.gov/gwastudies (February 2011) and literature search in pub med. We selected this phenotypes because it is representative selection among all complex traits and it meets the criteria proposed by Visscher et al: at least three GWAS papers published in journals with a 2010–2011 journal impact factor >9 and at least one paper containing 10 or more genome-wide significant loci. We found 19 GWA studies and examined whether the studies considered sex differences, and if yes, whether they found significant sex differences. (DOC) [file pone.0047371.s009.doc]

**Supplemental Table S8. Overview of all published GWA studies for Height based on the database published on** [**www.genome.gov/gwastudies**](http://www.genome.gov/gwastudies) **(February 2011) and literature search in pub med. We selected this phenotypes because it is representative selection among all complex traits and it meets the criteria proposed by Visscher et al: at least three GWAS papers published in journals with a 2010-2011 journal impact factor >9 and at least one paper containing 10 or more genome-wide significant loci. We found 19 GWA studies and examined whether the studies considered sex differences, and if yes, whether they found significant sex differences.**

| **Author** | **Year of publication** | **Phenotypes** | **Were sex differences explored?** | **Sex-specific result?** | **Conclusion: Diff genes M/F?** |
| --- | --- | --- | --- | --- | --- |
| Y.S. Cho *et al* | 2009 | Height, BMI,  pulse rate, systolic BP, diastolic BP, waist-hip ratio,  bone mineral density | Yes, in 11 top-hits SNPs in stage 1 GWAS and in stage 2 replication analyses | 1 SNP (associated with SBP) showed nominal statistical significance, but this sex-specific effect was not replicated.  No significant sex-heterogeneity at the height associated signals. | No |
| A. N’Diaye *et al* | 2011 | Height  (meta-analyses) | Yes, for 2 SNPs | No significant evidence of sex heterogeneity (P=.26 & P=.34, respectively). | No |
| C.L. Carty *et al* | 2012 | Height | No (replication analyses of 169 SNPs was carried out using sex- (and disease status-) specific height Z-scores) |  | NA |
| D.C. Croteau-Chonka *et al* | 2010 | Height, weight,  BMI,  waist circumference | No (the sample consisted of women only). |  | NA |
| J.Z. Liu *et al.* | 2010 | Height, BMI | No (sex and sex by SNPs interaction as covariates) |  | NA |
| A. Tonjes et al | 2009 | Height | 1 significant SNP was further considered in two independent cohorts | SNP was replicated in males (p=.049 & p=.022) not in females (p=.965 & p=.055). But no sex differences in the discovery sample | Inconclusive |
| A. Johansson *et al* | 2008 | Height | Linkage and GWA were run stratified for sex in 5 European samples and in combined sample | No sex-specific results in linkage or GWA. However, signal strength of the significant SNPs was different in males and females. | No |
| DF. Gudjartsson *et al* | 2008 | Height | Yes, effect sizes for the identified SNPs were compared for males and females | No significant differences between sexes (p-values>.002). | No |
| G. Lettre *et al* | 2008 | Height | Yes, effect sizes of 16 identified SNPs (combined P < 5X10^-6^) were compared for males and females in a replication sample. | No evidence for sex heterogeneity  (P > .05). | No |
| S. Sanna *et al* | 2008 | Height | 2 SNPs were considered | No evidence for sex heterogeneity | No |
| M.N. Weedon *et al.* | 2007 | Heigth | 1 SNP was considered | No evidence for sex heterogeneity | No |
| M.N. Weedon *et al.* | 2008 | Height | Yes, effect sizes of 20 SNPs were compared for males and females in stage 2 joint analyses. | Of the 20 significant loci, 1 SNP was found to have a significant greater effect in females than males (P=.01). | 1 SNP larger effect size in females. |
| H. Lango Allen | 2010 | Height | Yes, sex-specific analyses of the 180 associated signals  in the stage1 + stage2 samples | No differences between their effects in males compared to females (p-values>0.01) | No |
| HN Kim | 2010 |  |  |  |  |
| Y. Okada | 2010 | Height | No (sex as covariate) |  | NA |
| J.J. Kim | 2009 | Height (adults), idiopatic short stature (children) | No (sex as covariate) |  | NA |
| K Estrada | 2009 | Height | No (sex as covariate) |  | NA |
| N Soranzo | 2009 | Height, trunk length, hip axis length and femur length | Yes, sex-specific effects at the 17 validated height loci were investigated in 1 sample (the Rotterdam sample) | Significant sex-heterogeneity at 3 SNPs (p=.01, I^2^=85%; p=.002, I^2^=89% & p=.002. I^2^=89%, respectively); | Significant sex-heterogeneity at 3 SNPs (Nfemales=3,374; Nmales  =2,362) |
| SF Lei | 2008 | Height | No (sex as covariate) |  | NA |
